# Nano/Micro Metal‐Organic Framework‐Derived Porous Carbon with Rich Nitrogen Sites as Efficient Iodine Hosts for Aqueous Zinc‐Iodine Batteries

**DOI:** 10.1002/advs.202502563

**Published:** 2025-04-15

**Authors:** Yong Li, Xiaotian Guo, Shixian Wang, Wenzhuo Sun, Dianheng Yu, Nana Li, Huijie Zhou, Xiaoxing Zhang, Huan Pang

**Affiliations:** ^1^ School of Chemistry and Chemical Engineering Yangzhou University Yangzhou Jiangsu 225009 P. R. China; ^2^ State Key Laboratory of Coordination Chemistry Nanjing University Nanjing Jiangsu 210093 P. R. China; ^3^ Institute of Technology for Carbon Neutralization Yangzhou University Yangzhou Jiangsu 225127 P. R. China

**Keywords:** aqueous zinc‐iodine battery, nano/micro metal‐organic framework, porous carbon, practical application

## Abstract

Nowadays, the low cost, superior safety, and long durability of aqueous zinc–iodine (Zn–I_2_) batteries have garnered significant interest. Nevertheless, their practical use is limited by the inferior conductivity of iodine and the shuttle effect, resulting in suboptimal electrochemical behavior. In this work, a nano/micro zinc‐based metal‐organic framework (Zn‐MOF) featuring a cubic morphology is employed for obtaining porous nitrogen‐doped carbon (NC), which is reported as a cathode host for Zn–I_2_ batteries. Thanks to its porous architecture and high conductivity of NC, the optimized S3‐1000 material achieves high iodine loading and enables rapid electron/ion transport. More importantly, the adsorption experiments combined with density functional theory (DFT) calculations reveal that the graphitic‐N and pyridine‐N moieties within the carbon matrix synergistically serve as active anchoring sites for iodine species, suppress polyiodide shuttle effects and accelerate redox kinetics during iodine conversion. As a result, the I_2_@S3‐1000 cathode achieves the highest specific capacity and superior cycle stability over 10,000 cycles, while in‐situ characterization analysis confirms the reversible electrochemical mechanism. Soft pack battery and prototype flexible micro‐battery based on the I_2_@S3‐1000 cathode are also fabricated and show excellent flexibility. This study promotes the development of MOF‐derived carbon as iodine cathodes for advanced Zn–I_2_ batteries.

## Graphical abstract

A cubic zinc‐based metal‐organic framework derived (Zn‐MOF‐derived) porous nitrogen‐doped carbon host is engineered to immobilize iodine, leveraging its hierarchical porosity and rich nitrogen sites to suppress polyiodide shuttling and enhance redox kinetics in zinc–iodine (Zn–I_2_) batteries. The flexible battery prototypes demonstrate exceptional cycling stability and mechanical adaptability.

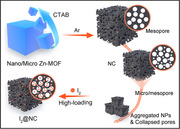

## 1 Introduction

Aqueous zinc‐iodine (Zn–I_2_) batteries show promise for large‐scale energy storage because of their long cyclability, environmentally friendly operation, and economical cost.^[^ ^1^, ^2^, ^3^ ^]^ Nevertheless, the inferior electrical conductivity of iodine and the shuttle effect hinder their practical use.^[^ ^4^, ^5^ ^]^ To address these issues, porous carbon‐based materials, such as carbon fibers, activated carbon, carbon nanotubes, and ordered mesoporous carbon have been efficient cathode hosts for Zn–I_2_ batteries.^[^ ^6^, ^7^, ^8^ ^]^ On the one hand, the introduction of carbon can improve the conductivity of iodine via physical adsorption.^[^ ^9^, ^10^ ^]^ On the other hand, the substantial porosity of porous carbon hosts is beneficial for diminishing the shuttle effect of iodine species (I_3_^−^ and I^−^).^[^ ^11^, ^12^, ^13^ ^]^ However, there remains the issue of the shuttle effect upon cycling, which results in reduced capacity and severe self‐discharge. Moreover, poor cycling performance stems from the weak physical adsorption of a nonpolar carbon framework host, which impedes its ability to address the polyiodide shuttle effect effectively. Therefore, appropriate host materials need to be crafted to reduce the shuttle effect through chemical interactions that exhibit strong binding energies.^[^ ^14^, ^15^ ^]^

Metal‐organic frameworks (MOFs) have garnered attention because of their large surface area (SSA), tunable pore size, and designable structures.^[^ ^16^, ^17^, ^18^ ^]^ Due to its vast surface area and porous structure, MOF‐derived carbon serves as an ideal host for iodine.^[^ ^19^ ^]^ Through precise pyrolysis condition optimization, MOF‐derived carbons can achieve tailored pore size distributions ranging from micropores to mesopores, which effectively accommodate iodine species while maintaining structural integrity.^[^ ^19^ ^]^ The incorporation of heteroatoms (N, S, P, and B) into porous carbon further enhances physical adsorption and promotes interaction with iodine species, creating a polar surface along with active sites that facilitate the iodine conversion, thereby boosting electrochemical performance.^[^ ^20^, ^21^ ^]^ Among various MOF precursors, demonstrate exceptional advantages in fabricating heteroatom‐doped porous carbons, owing to their structural stability during carbonization while facilitating the formation of desired porous architectures with uniform heteroatom distribution.^[^ ^22^ ^]^ This unique combination of structural and chemical properties makes MOF‐derived carbons ideal candidates for ideal iodine host materials in electrochemical applications.

Hence, we proposed Zn‐MOF‐derived porous nitrogen‐doped carbon materials (NC) as cathode hosts for aqueous Zn–I_2_ batteries. Hexadecyl trimethyl ammonium bromide (CTAB) as a surfactant and capping agent had a notable effect on the synthesis of Zn‐MOF nanocubes. The ordered porous NC, characterized by its large SSA and micro/mesopores, enables effective iodine loading/trapping and promotes efficient electron/ion transfer throughout cycling. Moreover, pyridinic‐N/graphitic‐N doping is highly beneficial for adsorbing iodine species and facilitating their conversion in Zn–I_2_ batteries. Benefiting from the above merits, the I_2_@S3‐1000 cathode exhibited superior long‐term cyclability with a discharge capacity of 112.4 mA h g^−1^ at the 10,000th cycle even at 2 A g^−1^ and the I_2_@S3‐1000‐based soft pack battery with superior mechanical flexibility exhibited a capacity of 172.9 mA h g^−1^ after 100 cycles. Besides, the establishment of the integrated micro‐battery demonstrated their potential for real‐scale applications.

## 2 Results and Discussion

**Figure 1:**
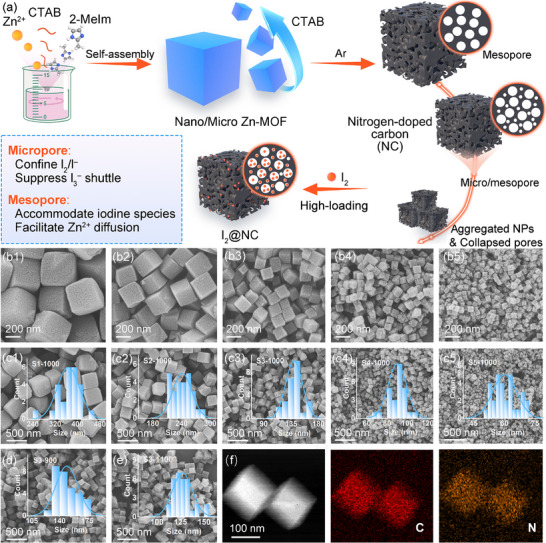
a) Schematic synthesis of cubic Zn‐MOF derived NC and I_2_@NC. SEM images and corresponding diameter size distributions of (b1,c1) S1‐1000; (b2,c2) S2‐1000; (b3,c3) S3‐1000; (b4,c4) S4‐1000; (b5,c5) S5‐1000. d,e) Diameter size distributions insert SEM images of S3‐900 and S3‐1100, respectively. f) The elemental mapping results of S3‐1000.

The schematic synthesis of cubic Zn‐MOF‐derived porous NC is presented in **Figure** **1**a. Zn‐MOF nanocubes named S1–S5 were formed by the coordination of the metal salts and 2‐Methylimidazole (2‐MeIm) under the regulation of CTAB. During crystal growth, the differential growth rates of crystal facets lead to the formation of cubic morphology, with the {100} facets exhibiting relatively faster growth rates.^[^ ^23^ ^]^ The amount of CTAB from 5 to 40 mg affected the size and structure of Zn‐MOF‐derived NC nanocubes As an end‐sealing agent, the hydrophobic long chain of CTAB was adsorbed on the surface of Zn‐MOF, preventing the growth of crystal nuclei and reducing the particle size. Figure 1b1–b5 showed the scanning electron microscopy (SEM) images of a series of the S1‐1000, S2‐1000, S3‐1000, S4‐1000, and S5‐1000 samples. As the amount of CTAB increased, the size of the S1‐1000–S5‐1000 samples decreased from 370 to 60 nm (Figure 1c1–c5).

The morphologies of S1‐1000 and S2‐1000 remained stable after calcination, despite their sizes being larger than 200 nm. In contrast, the S4‐1000 and S5‐1000 samples, with average diameters of 90 and 60 nm, respectively, partially collapsed after calcination. Therefore, considering the interplay between the decreased size and nanostructure integrity without significant aggregation, S3 was determined to be a suitable MOF precursor for obtaining S3‐1000 (average size:140 nm) as a cathode host (Figure S1, Supporting Information), which was also revealed by the performance later. Moreover, to investigate the influence of nitrogen species on their performance, S3‐900 and S3‐1100 samples with average diameters of ≈150 and 125 nm, respectively, were prepared through calcination at 900 and 1100 °C while maintaining their cubic structures (Figure 1d,e). The amorphous carbon structure of S3‐1000 was further studied by transmission electron microscopy (TEM), high‐resolution TEM (HRTEM) images, and the selected area electron diffraction (SAED) patterns (Figure S2, Supporting Information). The Zn ions emitted from the core of Zn‐MOFs underwent volatilization during the carbonization process, leaving ample micro/mesopores and nitrogen sources, thereby resulting in a porous NC framework.^[^ ^24^, ^25^ ^]^ The C and N elements are homogeneously distributed within the S3‐1000 sample (Figure 1f).

**Figure 2:**
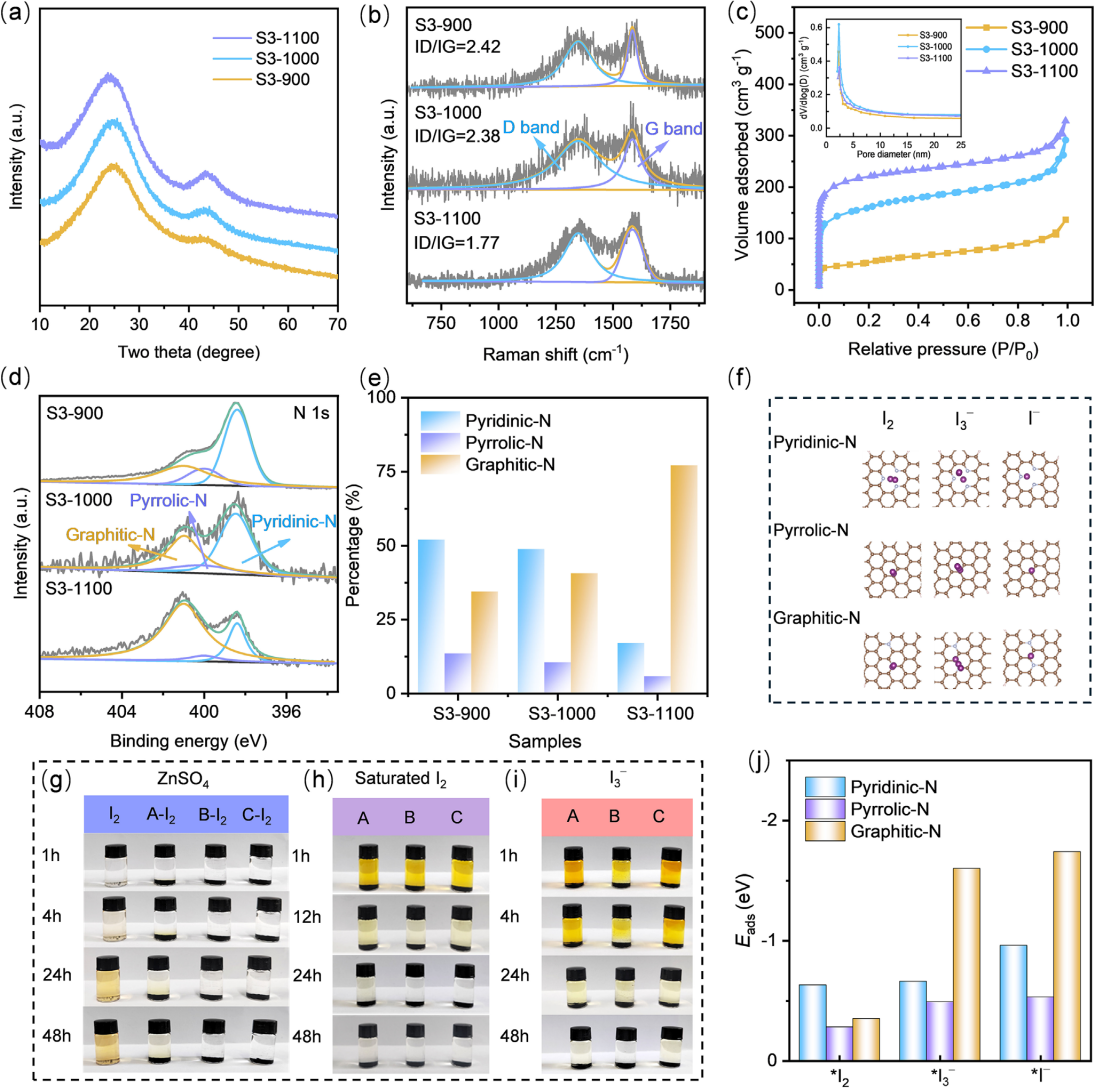
a,b) XRD patterns and Raman spectra of S3‐X (X = 900, 1000, 1100); c) SSAs and mesopore size distribution curves of the S3‐900, S3‐1000, and S3‐1100 samples; d) High‐resolution N 1s spectra of S3‐900, S3‐1000, and S3‐1100, respectively; e) Bar chart of the percentages of the nitrogen species; f) Model constructions showing the interaction of I_2_ and intermediates with different nitrogen species, respectively; g) Digital photos for the solubility in 2 m ZnSO_4_ solution (A: S3‐900, B: S3‐1000, C: S3‐1100). Digital photos of S3‐900, S3‐1000, and S3‐1100 in (h) saturated I_2_ and i) Zn(I_3_)_2_ solution, respectively. j) The DFT calculations of binding energies between iodine species and pyridine‐N, pyrrolic‐N, and graphitic‐N.

The diffraction peaks observed in the powder X‐ray diffraction (XRD) patterns for the S3 precursor at 7.3°, 10.3°, 12.7°, 14.7°, 16.4°, 18.0° and 26.6° (Figure S3, Supporting Information), are attributed to ZIF‐8.^[^ ^26^ ^]^ The XRD patterns of S3‐900, S3‐1000, and S3‐1100 displayed broad peaks at ≈24.6° and 43.5° (**Figure** **2**a), which are indicative of amorphous carbon. Furthermore, the Raman spectra in Figure 2b displayed two peaks centered at ≈1348 cm^−1^ (D band) and 1587 cm^−1^ (G band). The increasing pyrolysis temperature led to the gradual increase in ID/IG ratios for S3‐X (X = 900, 1000, 1100). Specifically, the ID/IG values of the S3‐900, S3‐1000, and S3‐1100 were measured at 2.42, 2.38, and 1.77, respectively. The lower ID/IG ratio suggests enhanced electronic conductivity, which is conducive to enhancing performance.^[^ ^27^ ^]^ As shown in Figure S4 (Supporting Information), the 43.7 wt.% iodine loading of I_2_@S3‐1000 is displayed by thermogravimetric analysis (TGA). Besides, the Nitrogen adsorption–desorption isotherms demonstrated that the S3‐X (X = 900, 1000, 1100) samples endow a plentiful porous structure consisting of micropores and mesopores, as evidenced by the sharp increase in the isotherm at relatively low pressures (P/P_0_ < 0.1), allowing for efficient iodine adsorption.^[^ ^28^, ^29^ ^]^ The S3‐1000 sample had a SSA of 538.2 m^2^ g^−1^ and an average pore size of 3.3 nm (Figure 2c), whereas the S3‐900 and S3‐1100 samples had SSA values of 188.9 and 712.5 m^2^ g^−1^, and average pore sizes of 4.3 and 2.8 nm, respectively. The micropore volumes of S3‐X (X = 900, 1000, 1100) were 0.079, 0.242, and 0.337, respectively (Figure S5, Supporting Information). The micropores can enhance iodine conversion kinetics while suppressing the I_3_⁻ shuttle effect, and provide abundant active sites through their high SSA for efficient I_2_ adsorption and conversion.^[^ ^26^ ^]^

To explore the chemical interactions and elemental states, X‐ray photoelectron spectroscopy (XPS) analysis of I_2_@S3‐X (X = 900, 1000, 1100) was carried out. The full XPS spectra of S3‐X revealed the existence of C, N, and O elements, implying that N atoms were successfully integrated into the carbon matrix during carbonization (Figure S6, Supporting Information). The C 1s spectrum of S3‐X was divided into C─C/C═C (284.8 eV), C─N (285.8 eV), and C─O/C═O (286.8 eV) (Figure S7, Supporting Information). The relative nitrogen content of S3‐1000 (14.75%) is comparable to that of S3‐900 (15.33%), whereas the nitrogen ratio of S3‐1100 decreases to 11.16% because of the thermal decomposition of unstable nitrogen moieties at higher temperatures. To a certain extent, porous carbon with high N‐content may decrease the effective SSA and porous carbon with low N‐content destroys the framework as an iodine host, affecting their performance.^[^ ^15^ ^]^ Hence, constructing a well‐suited N‐doping configuration in porous carbon is crucial for optimizing its role as an iodine‐loading host in high‐performance Zn–I_2_ batteries. Figure 2d shows the N 1s spectra were fitted into three peaks of pyridinic‐N, pyrrolic‐N, and graphitic‐N in the S3‐X (X = 900, 1000, 1100), respectively.^[^ ^30^ ^]^ The ratios of the different nitrogen species are presented in Figure 2e and Table S1 (Supporting Information).

To better understand the effect of nitrogen species on adsorption durability and capability for S3‐X (X = 900, 1000, 1100) products, the experiments of electrolyte immersion and the adsorption of iodine species including saturated I_2_ and I_3_^−^ were conducted. The colors of I_2_ and I_2_@S3‐900 solutions turned light‐brown after 4 h in the 2 m ZnSO_4_ electrolyte, while the color in I_2_@S3‐1000 and I_2_@S3‐1100 did not show obvious change even after 48 h (Figure 2g). The graphitic‐N species in S3‐1000 and S3‐1100 had a critical effect on the less self‐discharge and superb stability of iodine cathodes to some extent.^[^ ^25^ ^]^ In addition, the color of S3‐1000 and S3‐1100 completely turned colorless after 48 h in saturated I_2_ solution, whereas S3‐900 did not turn colorless, indicating weaker adsorption capability of S3‐900 (Figure 2h). Similarly, the solution of the S3‐1000 sample became completely transparent after 48 h while the solution of the S3‐900 sample with a higher ratio of pyridine‐N exhibited a light‐yellow color in the I_3_^−^ solution (Figure 2i), revealing that both pyridine‐N and graphitic‐N play a critical effect on the adsorption of the iodine species including I_2_ and I_3_^−^ ions.^[^ ^31^, ^32^ ^]^

Based on density functional theory (DFT) calculations, the graphitic‐N species exhibit more negative adsorption energies toward I^−^ (−1.74 eV) and I_3_⁻ (−1.60 eV), followed by pyridinic‐N and pyrrolic‐N (Figure 2f,j). The stability of S3‐1000 and S3‐1100, where minimal color change in electrolyte immersion and rapid iodine adsorption indicate robust chemical confinement of I^−^ and I_3_⁻. The superior performance of graphitic‐N arises from its delocalized π‐electron system and planar coordination geometry, which enhance charge transfer and orbital hybridization with iodine species. In contrast, the lone‐pair electrons and non‐planar configuration of pyrrolic‐N weaken its interaction with iodine, resulting in slower adsorption kinetics of S3‐900. Besides, the pyridinic‐N species exhibits the strongest adsorption energy toward I_2_, indicating that pyridinic‐N is crucial for the initial chemical binding and capture of iodine molecules.^[^ ^33^ ^]^ Notably, the intermediate adsorption strength of pyridinic‐N synergizes with graphitic‐N in S3‐1000 and S3‐1100, enhancing iodine confinement through edge‐site reactivity and π‐electron delocalization. The adsorption experiments combined with DFT results demonstrated that graphitic‐N is the dominant contributor to adsorption durability and pyridinic‐N is a complementary active site for iodine conversion.

**Figure 3:**
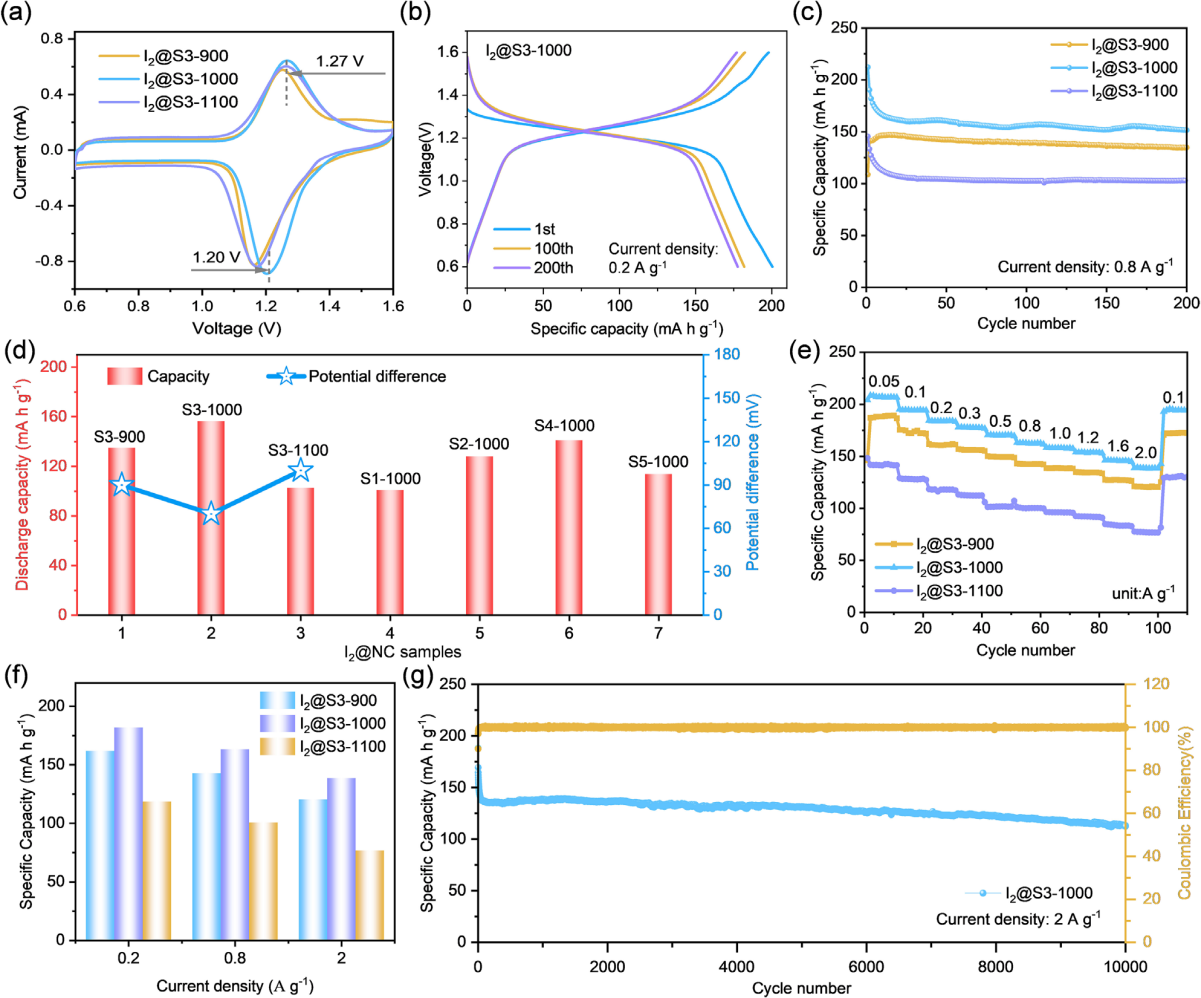
a) CV curves of I_2_@S3‐900, I_2_@S3‐1000, and I_2_@S3‐1100 at 0.2 mV s^−1^; b) GCD curves of I_2_@S3‐1000 at 0.2 A g^−1^; c) Cycling performance of I_2_@S3‐900, I_2_@S3‐1000, and I_2_@S3‐1100 at 0.8 A g^−1^; d) Bar chart of the performance of a series of I_2_@NC samples at 0.8 A g^−1^ for 200 cycles; e,f) Rate performance and bar chart of I_2_@S3‐900, I_2_@S3‐1000, and I_2_@S3‐1100 at various current densities; g) Long‐term cyclability of I_2_@S3‐1000 at 2 A g^−1^ for 10,000 cycles.

In aqueous Zn–I_2_ batteries, the electrochemical performance of the I_2_@S3‐X (X = 900, 1000, 1100) cathodes was assessed. The cyclic voltammetry (CV) tests of the I_2_@S3‐X cathodes demonstrated the redox reactions in the voltage range of 0.6–1.6 V (**Figure** **3**a). The oxidation‐reduction peaks of 1.17/1.26, 1.20/1.27, and 1.17/1.27 V, were observed in the I_2_@S3‐900, I_2_@S3‐1000, and I_2_@S3‐1100 cathodes, respectively. Obviously, the I_2_@S3‐1000 cathode exhibited the smallest polarization voltage of 70 mV, compared to the I_2_@S3‐900 cathode (90 mV) and the I_2_@S3‐1100 cathode (100 mV), illustrating the lower polarization and boosted redox kinetics of the I_2_@S3‐1000 cathode. The galvanostatic charge/discharge (GCD) curves of I_2_@S3‐1000 showed initial discharge/charge capacities of 200.5/198.1 mA h g^−1^ at 0.2 A g^−1^, respectively (Figure 3b). Additionally, I_2_@S3‐1000 delivered a specific capacity of 177.7 mA h g^−1^ at 0.2 A g^−1^ at the 200th cycle (Figure S8, Supporting Information). The cycling performance of the I_2_@S3‐900, I_2_@S3‐1000, and I_2_@S3‐1100 cathodes were measured at 0.8 A g^−1^ (Figure 3c). The I_2_@S3‐1000 cathode achieved a reversible capacity of 156.3 mA h g^−1^ after 200 cycles, higher than the I_2_@S3‐900, I_2_@S3‐1100, I_2_@S1‐1000, I_2_@S2‐1000, I_2_@S4‐1000, and I_2_@S5‐1000 cathodes (Figure 3d; Figure S9, Supporting Information), indicating that I_2_@S3‐1000 possessed better electrochemical activity. The measured discharge capacity of the pristine S3‐1000 electrode without iodine loading remained at around 7 mA h g⁻¹ (Figure S10, Supporting Information), confirming that the S3‐1000 host material itself contributes minimally to the charge storage capacity.

Figure 3e shows the rate performance of I_2_@S3‐900, I_2_@S3‐1000, and I_2_@S3‐1100 at current densities of 0.05–2 A g^−1^. The I_2_@S3‐1000 cathode displayed discharge capacities of 208.5 and 138.6 mA h g^−1^ at 0.05 and 2 A g^−1^, respectively. I_2_@S3‐1000 achieved a discharge capacity of 193.2 mA h g^−1^ upon returning the current density to 0.1 A g^−1^. Nevertheless, the discharge capacities of I_2_@S3‐900 and I_2_@S3‐1100 can only achieve 120.5 and 77.4 mA h g^−1^ at 2 A g^−1^, respectively, which is much smaller than that of I_2_@S3‐1000 (Figure 3f). It indicated the remarkable rate capability of the I_2_@S3‐1000 cathode resulting from the superior conductivity of Zn‐MOF‐derived porous NC. Besides, the graphitic‐N species can create robust chemical adsorption sites for iodine species, thereby significantly restricting their diffusion in the electrolyte.^[^ ^11^ ^]^ Figures S11 and S12 (Supporting Information) showed the GCD curves of I_2_@S3‐900, I_2_@S3‐1000, and I_2_@S3‐1100 at different current densities. The GCD curves of the I_2_@S3‐1000 cathode show the voltage plateaus within a range of 1.1–1.4 V at different current densities and the smallest voltage polarization compared to those of I_2_@S3‐900 and I_2_@S3‐1100. The symmetrical charge‐discharge platform of I_2_@S3‐1000 at different current densities also revealed its good redox reversibility. Additionally, the long‐term cyclability of the I_2_@S3‐1000 cathode was further investigated at 2 A g^−1^ (Figure 3g). A discharge capacity of 112.4 mA h g^−1^ after 10,000 cycles and the capacity decay rate is only 0.0040% per cycle, indicating its excellent reversibility and long cyclability.

**Figure 4:**
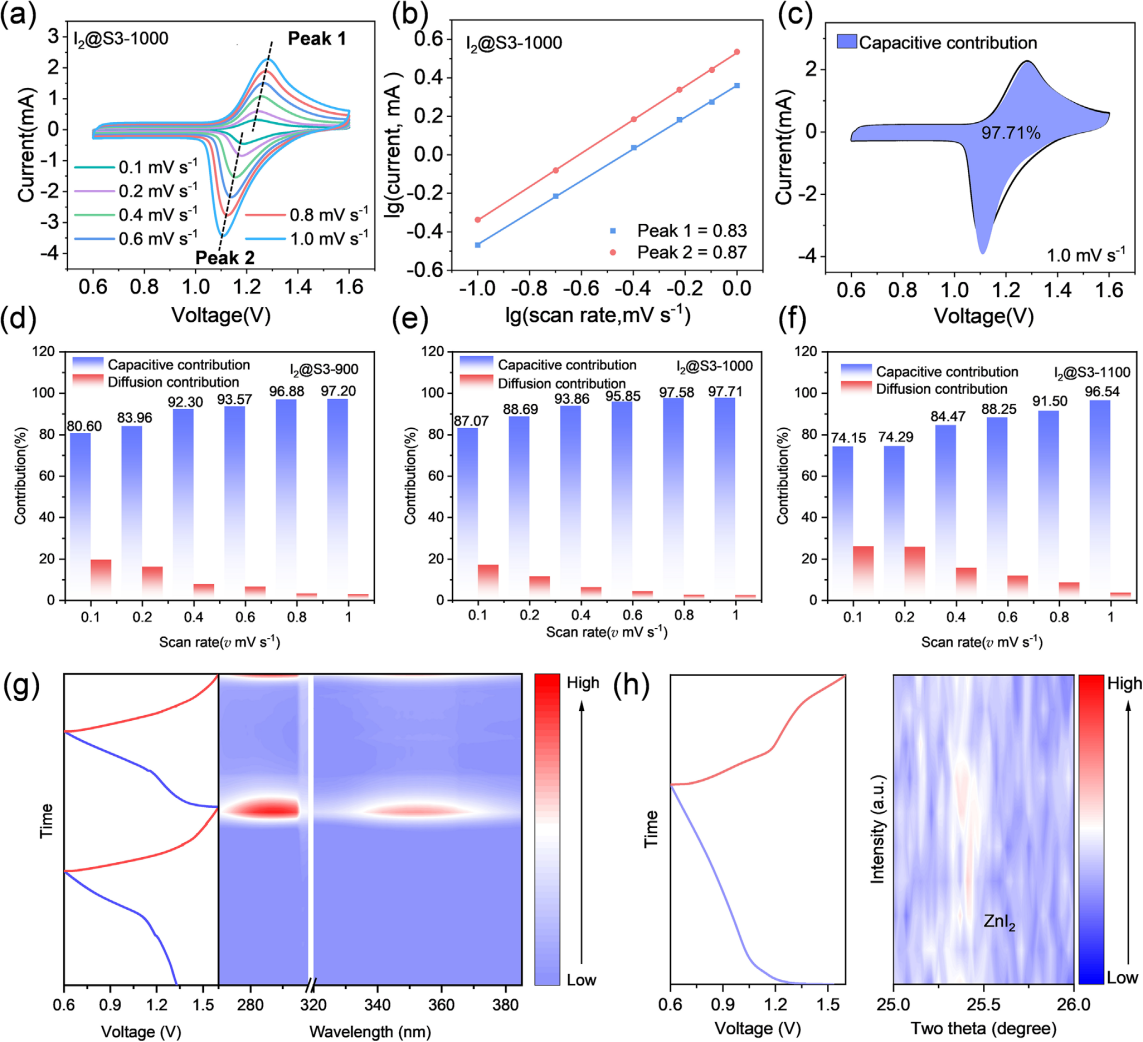
a) CV curves of I_2_@S3‐1000 at different scan rates; b) The linear fitting plots of the b values from CV curves of I_2_@S3‐1000; c) The capacitive contribution of I_2_@S3‐1000 at scan rates of 1.0 mV s^−1^; d–f) Percentage of capacitive contributions of I_2_@S3‐900, I_2_@S3‐1000, and I_2_@S3‐1100 at different scan rates; g) In situ UV–vis spectra of I_2_@S3‐1000; h) In situ XRD analysis of I_2_@S3‐1000.

To study the redox reaction process of the I_2_@NC cathodes, the CV curves of the I_2_@S3‐900, I_2_@S3‐1000, and I_2_@S3‐1100 electrodes were measured at different scan rates, as displayed in **Figure** **4**a and Figure S13 (Supporting Information). As the scan rate increased, the voltage polarization of I_2_@S3‐1000 increased. The current (*i*) is related to the scan rate (*v*) according to the equation *i = av^b^*, with *a* being a variable parameter, and *b* calculated from the slope of the log *i*‐log *v* curve (Figure 4b; Figure S14, Supporting Information).^[^ ^34^ ^]^ The b values corresponding to the reduction and oxidation peaks for the I_2_@S3‐1000 cathode in the CV curves were 0.83 and 0.87, respectively, suggesting the capacity behavior of the Zn–I_2_ battery comes from both diffusion‐dominated and surface capacitive processes. The pseudocapacitive contribution is further calculated by the formula: *i = k*_1_ *v + k*_2_ *v* ^1/2^, where *k*_1_ *v* is the pseudocapacitive contribution and *k*_2_ *v* ^1/2^ accounts for the diffusion‐controlled process.^[^ ^35^ ^]^ The capacitive contributions of the I_2_@S3‐900, I_2_@S3‐1000, and I_2_@S3‐1100 cathodes increased with the increase of the various scan rates (Figure 4c–f; Figures S15–S17, Supporting Information). I_2_@S3‐1000 demonstrated enhanced kinetics with a capacitive contribution of 97.71%, whereas the capacitive contributions of I_2_@S3‐900 and I_2_@S3‐1100 reached 97.20% and 96.54% at 1.0 mV s^−1^, respectively. Furthermore, the in situ UV–vis experiment was used to explore the electrochemical mechanism of the optimized I_2_@S3‐1000 sample in the 2 m ZnSO_4_ electrolyte. As shown in Figure 4g, two obvious peaks appeared at 287.5 and 352.1 nm corresponding to I_3_^−^ in the electrolyte during the charging–discharging process.^[^ ^36^ ^]^ The in situ XRD patterns of the I_2_@S3‐1000 cathode during the charge–discharge process of Zn–I_2_ batteries were also presented. Notably, the peak observed at 25.4° in the contour plot corresponded to the formation of ZnI_2_ (Figure 4h), reflecting the mechanism of Zn–I_2_ batteries.^[^ ^37^ ^]^

**Figure 5:**
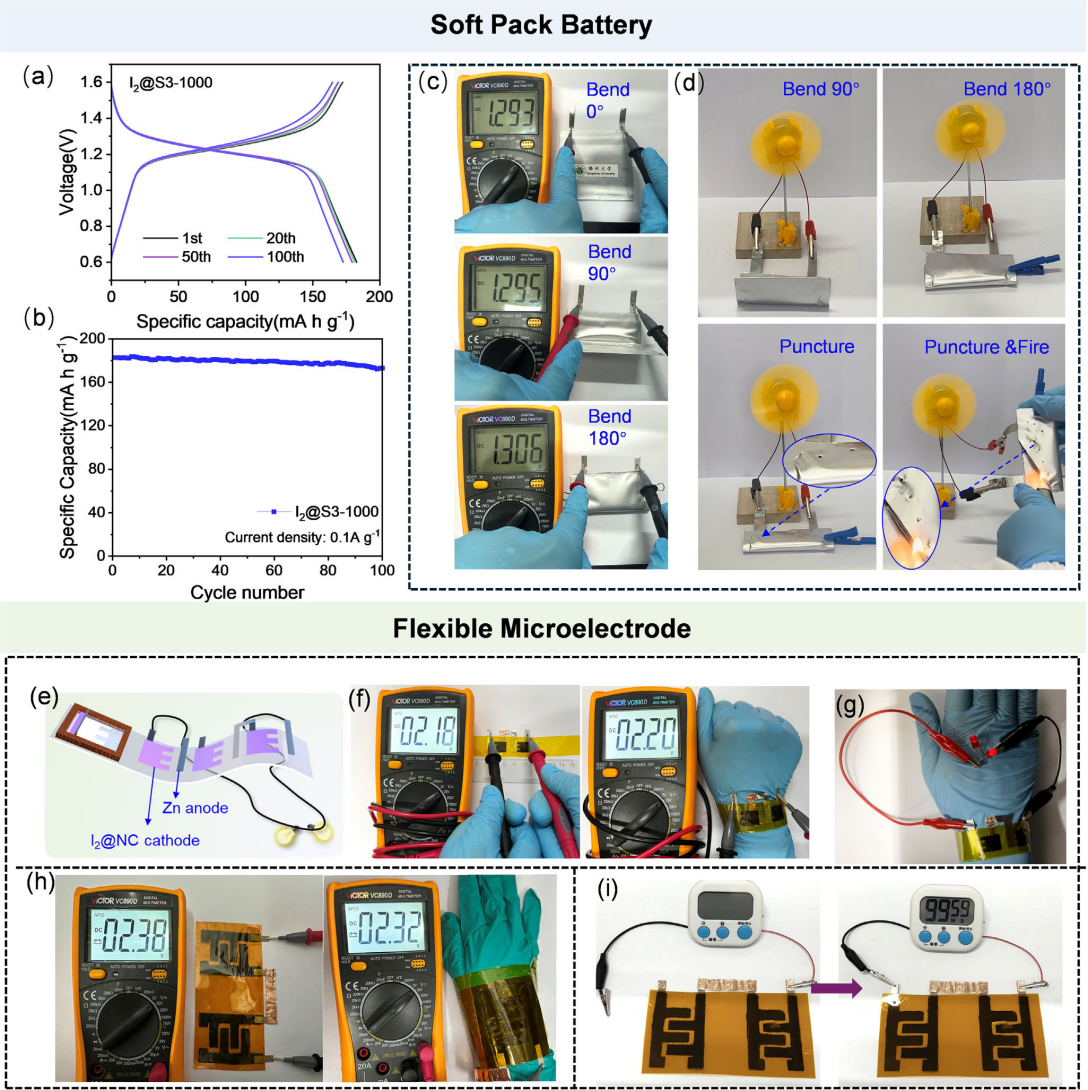
a) GCD curves and b) cyclability of the soft pack battery based on I_2_@S3‐1000 cathode at 0.1 A g^−1^; c,d) Voltage stability of a single soft pack battery and driving a small electric fan powered by a soft pack battery at various conditions; e) The diagram showing the flexible microelectrode; f) Voltage stability of the two microelectrodes in series in flat and twisting states; g) The photograph of the microelectrodes with two red LEDs; h) Voltage stability of the two integrated micro‐batteries in series in flat and twisting states; i) Photograph showing that the two integrated micro‐batteries in series can power the timer.

To verify the prospective application of the I_2_@S3‐1000 cathode in the electrochemical energy storage devices, the corresponding soft pack battery and flexible micro‐battery were assembled. **Figure** **5**a describes the GCD curves of the soft pack battery based on I_2_@S3‐1000 cathode with discharge/charge capacities of 182.8 and 172.4 mA h g^−1^ at 0.1 A g^−1^. Figure 5b presents that the as‐fabricated Zn–I_2_ battery has a specific capacity of 172.9 mA h g^−1^ at the 100th cycle. In the subsequent voltage stability testing experiment, the S3‐1000‐based soft pack battery was bent at various angles ranging from 0° to 180° and had a stable voltage of around 1.29 V, attesting the extraordinary flexibility (Figure 5c). The soft pack battery can continuously power a small electric fan under various bending angles, puncture, and fire conditions (Figure 5d). Furthermore, a screen‐printing technology was employed to assemble the I_2_@S3‐1000 cathode and integrated micro‐battery based on the I_2_@S3‐1000 cathode//Zn anode for wearable applications.^[^ ^38^, ^39^, ^40^ ^]^ The assembly process of the I_2_@S3‐1000 microelectrode that was conveniently incorporated onto a bendable polyimide substrate is illustrated in Figure 5e. The voltage of two microelectrodes in series maintained stable output voltages in both flat and bent states (Figure 5f,h). Impressively, two series‐connected microelectrodes that were wrapped around the wrist could adequately power two red light‐emitting diodes (LEDs) in parallel (Figure 5g). The two integrated micro‐batteries in series could continuously power the timer for ≈100 min (Figure 5i), validating the integrated design for wearable energy systems.

## 3 Conclusion

In summary, porous N‐C materials were synthesized from nano/micro Zn‐MOF precursors with CTAB critically regulating the cubic morphology and size control. The abundant pyridine‐N and graphitic‐N sites provide active sites for robust chemical interaction with iodine species and enhance electrical conductivity, thereby resulting in satisfactory electrochemical performance. As a result, the I_2_@S3‐1000 cathode exhibited an excellent long‐term cyclability with a discharge capacity of 112.4 mA h g^−1^ after 10,000 cycles even at 2 A g^−1^. Moreover, the practical applications including soft pack batteries and wearable micro‐batteries show exceptional performance, becoming a promising candidate for flexible power sources. This work provides the fundamental principle for the rational design of MOF‐derived heteroatom‐doped porous carbon as efficient iodine hosts, facilitating the development of aqueous Zn–I_2_ batteries.

## Conflict of Interest

The authors declare no conflict of interest.

## Supporting information



Supporting Information

## Data Availability

The data that support the findings of this study are available in the supplementary material of this article.

## References

[1] Q. Fu , W. Zhang , X. Liu , Y. Liu , Z. Lei , M. Zhang , H. Qu , X. Xiao , X. Zhong , Z. Liu , P. Qin , J. Yang , G. Zhou , J. Am. Chem. Soc. 2024, 146, 34950. 39632451 10.1021/jacs.4c14645

[2] C. Xie , C. Wang , Y. Xu , T. Li , Q. Fu , X. Li , Nat. Energy 2024, 9, 714.

[3] T. Liu , C. Lei , H. Wang , C. Xu , W. Ma , X. He , X. Liang , Sci. Bull. 2024, 69, 1674. 10.1016/j.scib.2024.02.014 38395648

[4] L. Ma , G. Zhu , Z. Wang , A. Zhu , K. Wu , B. Peng , J. Xu , D. Wang , Z. Jin , Nano Lett. 2023, 23, 5272. 37260235 10.1021/acs.nanolett.3c01310

[5] R.‐H. Wang , W. Wang , Y.‐Z. Zhang , W. Hu , L. Yue , J.‐H. Ni , W.‐Q. Zhang , G. Pei , S. Yang , L.‐F. Chen , Angew. Chem., Int. Ed. 2024, 64, 202417605. 10.1002/anie.202417605 39468954

[6] C. Bai , F. Cai , L. Wang , S. Guo , X. Liu , Z. Yuan , Nano Res. 2018, 11, 3548.

[7] Z. Li , W. Cao , T. Hu , Y. Hu , R. Zhang , H. Cui , F. Mo , C. Liu , C. Zhi , G. Liang , Angew. Chem., Int. Ed. 2024, 63, 202317652. 10.1002/anie.202317652 38086771

[8] F. Zhang , Q. Meng , J. Qian , J. Chen , W. Dong , K. Chen , Y. Cui , S. X. Dou , L. Chen , Angew. Chem., Int. Ed. 2025, 137, 202425487. 10.1002/anie.202425487 39853910

[9] M. Cui , H. Zhao , D. Yin , N. Gao , Y. Zhang , L. Zhao , Y. Wei , M. Liu , K. Xi , S. Ding , Energy Storage Mater. 2024, 69, 103372.

[10] J. He , H. Hong , S. Hu , X. Zhao , G. Qu , L. Zeng , H. Li , Nano Energy 2024, 119, 109096.

[11] Z. Bai , G. Wang , H. Liu , Y. Lou , N. Wang , H. K. Liu , S. Dou , Chem. Sci. 2024, 15, 3071. 38425533 10.1039/d3sc06150g PMC10901483

[12] Z. Liu , F. Feng , W. Feng , G. Wang , B. Qi , M. Gong , F. Zhang , H. Pang , Energy Environ. Sci. 2025, 18, 1929.

[13] P. Bao , L. Cheng , X. Yan , X. Nie , X. Su , H. Wang , L. Chen , Angew. Chem., Int. Ed. 2024, 63, 202405168. 10.1002/anie.202405168 38668683

[14] Y. Qu , J. Qian , F. Zhang , Z. Zhu , Y. Zhu , Z. Hou , Q. Meng , K. Chen , S. X. Dou , L. Chen , Adv. Mater. 2025, 37, 2413370. 10.1002/adma.202413370 39564705

[15] J. Xu , Z. Huang , H. Zhou , G. He , Y. Zhao , H. Li , Energy Storage Mater. 2024, 72, 103596.

[16] X. Zhuang , S. Zhang , Y. Tang , F. Yu , Z. Li , H. Pang , Coord. Chem. Rev. 2023, 490, 215208.

[17] G. Zhang , Y. Lu , Y. Yang , H. Yang , Z. Yang , S. Wang , W. Li , Y. Sun , J. Huang , Y. Luo , H. Y. Chen , Y. F. Liao , H. Ishii , S. Gull , M. Shakouri , H. G. Xue , Y. Hu , H. Pang , J. Am. Chem. Soc. 2024, 146, 16659.

[18] S. Zheng , Y. Sun , H. Xue , P. Braunstein , W. Huang , H. Pang , Natl. Sci. Rev. 2022, 9, nwab197. 35958682 10.1093/nsr/nwab197 PMC9362764

[19] H. Yu , Z. Wang , R. Zheng , L. Yan , L. Zhang , J. Shu , Angew. Chem., Int. Ed. 2023, 135, 202308397. 10.1002/anie.202308397 37458970

[20] J. Ma , M. Liu , Y. He , J. Zhang , Angew. Chem., Int. Ed. 2021, 60, 12636. 10.1002/anie.202009871 32939916

[21] H. Chen , X. Li , K. Fang , H. Wang , J. Ning , Y. Hu , Adv. Energy Mater. 2023, 13, 2302187.

[22] Z. Qiu , Y. Li , Y. Gao , Z. Meng , Y. Sun , Y. Bai , N. Suen , H. Chen , Y. Pi , H. Pang , Angew. Chem., Int. Ed. 2023, 62, 202306881. 10.1002/anie.202306881 37389975

[23] J. Troyano , A. Carné‐Sánchez , C. Avci , I. Imaz , D. Maspoch , Chem. Soc. Rev. 2019, 48, 5534. 31664283 10.1039/c9cs00472f

[24] L. Chai , X. Wang , Y. Hu , X. Li , S. Huang , J. Pan , J. Qian , X. Sun , Adv. Sci. 2022, 9, 2105063. 10.1002/advs.202105063 PMC9685461 36181364

[25] N. Li , Z. Yang , Y. Li , D. Yu , T. Pan , Y. Chen , W. Li , H. Xu , X. Guo , H. Pang , Adv. Energy Mater. 2024, 14, 2402846.

[26] X. Guo , H. Xu , Y. Tang , Z. Yang , F. Dou , W. Li , Q. Li , H. Pang , Adv. Mater. 2024, 36, 2408317. 10.1002/adma.202408317 39081106

[27] X. Guo , H. Xu , W. Li , Y. Liu , Y. Shi , Q. Li , H. Pang , Adv. Sci. 2023, 10, 2206084. 10.1002/advs.202206084 PMC9896072 36470654

[28] C. Guo , Y. Cao , Y. Gao , C. Zhi , Y. X. Wang , Y. Luo , X. J. Yang , X. Luo , Adv. Funct. Mater. 2024, 34, 2314189.

[29] S. Zheng , Q. Li , H. Xue , H. Pang , Q. Xu , Natl. Sci. Rev. 2020, 7, 305. 34692046 10.1093/nsr/nwz137 PMC8288962

[30] X. Guo , W. Li , Q. Zhang , Y. Liu , G. Yuan , P. Braunstein , H. Pang , Chem. Eng. J. 2022, 432, 134413.

[31] X. Wang , J. Yang , S. Liu , S. He , Z. Liu , X. Che , J. Qiu , Small 2024, 20, 2305508. 10.1002/smll.202305508 37670540

[32] Z. Gong , C. Song , C. Bai , X. Zhao , Z. Luo , G. Qi , X. Liu , C. Wang , Y. Duan , Z. Yuan , Sci. China Mater. 2023, 66, 556.

[33] W. Li , H. Xu , H. Zhang , F. Wei , L. Huang , S. Ke , J. Fu , C. Jing , J. Cheng , S. Liu , Nat. Commun. 2023, 14, 5235. 37640714 10.1038/s41467-023-40969-5 PMC10462634

[34] G. Yuan , Y. Su , X. Zhang , B. Gao , J. Hu , Y. Sun , W. Li , Z. Zhang , M. Shakouri , H. Pang , Natl. Sci. Rev. 2024, 11, 4. 10.1093/nsr/nwae336 PMC11487576 39430066

[35] J. Kang , C. Wang , Z. Liu , L. Wang , Y. Meng , Z. Zhai , J. Zhang , H. Lu , Energy Storage Mater. 2024, 68, 103367.

[36] Y. Lyu , J. A. Yuwono , P. Wang , Y. Wang , F. Yang , S. Liu , S. Zhang , B. Wang , K. Davey , J. Mao , Z. Guo , Angew. Chem., Int. Ed. 2023, 62, 202303011. 10.1002/anie.202303011 36949029

[37] W. Li , L. Huang , H. Zhang , Y. Wu , F. Wei , T. Zhang , J. Fu , C. Jing , J. Cheng , S. Liu , Matter 2023, 6, 2312.

[38] X. Cai , Y. Liu , J. Zha , F. Tan , B. Zhang , W. Yan , J. Zhao , B. Lu , J. Zhou , C. Tan , Adv. Funct. Mater. 2023, 33, 2303009.

[39] X. Jin , L. Song , C. Dai , Y. Xiao , Y. Han , X. Li , Y. Wang , J. Zhang , Y. Zhao , Z. Zhang , N. Chen , L. Jiang , L. Qu , Adv. Mater. 2022, 34, 2109450. 10.1002/adma.202109450 35139262

[40] K. K. Sonigara , J. Zhao , H. K. Machhi , G. Cui , S. S. Soni , Adv. Energy Mater. 2020, 10, 2001997.

