# Supplementary material for: Nano/Micro Metal‐Organic Framework‐Derived Porous Carbon with Rich Nitrogen Sites as Efficient Iodine Hosts for Aqueous Zinc‐Iodine Batteries
Source: Adv Sci (Weinh). 2025 Apr 15;12(26):2502563. doi: 10.1002/advs.202502563 (PMC12245103; doi:10.1002/advs.202502563)
Supplement: Supplementary file 1 — Supporting Information [file ADVS-12-2502563-s001.docx]

**Supporting Information**

**Nano/Micro Metal-Organic Framework-Derived Porous Carbon with Rich Nitrogen Sites as Efficient Iodine Hosts for Aqueous Zinc-Iodine Batteries**

*Yong Li^1^, Xiaotian Guo^1,*^, Shixian Wang^1^, Wenzhuo Sun^1^,* *Dianheng Yu^1^, Nana Li^1^, Huijie Zhou^3^, Xiaoxing Zhang^1^, and Huan Pang^1,2,*^*

^1^Mr. Y. Li, Dr. X. Guo, Mrs. S. Wang, Mr. W. Sun, Mrs. D. Yu, Mrs. N. Li, Mr. X. Zhang, Prof. H. Pang, School of Chemistry and Chemical Engineering, Yangzhou University, Yangzhou, Jiangsu, 225009, P. R. China, Email: panghuan@yzu.edu.cn

^2^Prof. H. Pang, State Key Laboratory of Coordination Chemistry, Nanjing University, Nanjing, Jiangsu, 210093, P. R. China

^3^Dr. H. Zhou, Institute of Technology for Carbon Neutralization, Yangzhou University, Yangzhou, Jiangsu, 225127, P. R. China

**Contents**

**1. Sample preparation**

**2. Sample characterization**

**3. Electrochemical characterization**

**4. Adsorption Experiments**

**5.** **Computational details**

**6. Supplementary Figures**

**7. Supplementary Tables**

**References**

**1.Sample preparation**

**1.1 Materials and reagents**

Zinc nitrate hexahydrate (Zn(NO_3_)_2_·6H_2_O, ≥99.0%) was purchased from Shanghai Sinopharm Chemical Reagent, co. ltd. 2-methylimidazole (99.0%) and iodine (I_2_, 99.99%) were purchased from Shanghai Aladdin reagent co. Ltd. All reagents used were used as received without further purification.

**1.2.1 Preparation of** **cubic Zn-MOF**

Cubic Zn-MOF nanocrystals were prepared by one-pot coprecipitation procedure. Briefly, 2-MeIm (11.3 g) was first dissolved in deionized water (164 mL) to form a clear aqueous solution. Then, 36 mL of aqueous solution containing CTAB (5 mg) and Zn (NO_3_)_2_·6H_2_O (725 mg) was rapidly added to above solution and stirred for 60 min to form Zn-MOF. Finally, the Zn-MOF was centrifuged at 10,000 rpm, and the obtained white-colored Zn-MOF precipitate was washed with ethanol several times and finally dried at 60 °C for 12 h. The Zn-MOF product was named as S1. When 5 mg of CTAB was replaced by 10 mg, 20 mg, 30 mg, and 40 mg, the corresponding products were named as S2–S5, respectively.

**1.2.2 Preparation of Zn-MOF-derived NC**

S1 was transferred into a corundum boat and placed in a tube furnace. The S1 powder was heated to 1000 °C with a heating rate of 5 °C min^–1^ and held for 2 h under Ar atmosphere and then naturally cooled to room temperature. Finally, the product was collected directly used without further treatment, which was named as S1-1000. Similarly, S2-1000, S3-1000, S4-1000, and S5-1000 powders were prepared by pyrolyzing the precursors of S2–S5 at a pyrolysis temperature of 1000 °C, respectively. S3-900 and S3-1100 were prepared by pyrolyzing S3 at pyrolysis temperatures of 900 °C and 1100 °C, respectively.

**1.2.3 Preparation of I_2_@NC composites**

Take I_2_@S3-1000 as an example, I_2_ and S3-1000 powders in a weight ratio of 1.5:1 was mixed, grinded, and placed in a sealed Teflon liner. The sealed Teflon liner was heated at 130 °C for 5 h and the mixture was named as I_2_@S3-1000.

**2. Sample characterization**

A series of the products were tested by X-ray diffraction (XRD) on a Bruker D8 Advanced X-ray Diffractometer (Cu-Kα radiation: λ=0.15406 nm) for the phase analysis. Scanning electron microscope (SEM, Zeiss_Supra55) was employed for studying the morphology of the samples at an acceleration voltage of 5.0 kV. High-resolution transmission electron microscopy (HRTEM) images and energy dispersive X-ray spectroscopy mapping images were captured on a Tecnai G2 F30 transmission electron microscopy at an acceleration voltage of 300 kV. X-ray photoelectron spectroscopy (XPS) tests were conducted on a Thermo Scientific ESCALAB 250 apparatus. Additionally, Brunauer-Emmett-Teller (BET) test was conducted on the Autosorb IQ3 instrument. Thermogravimetry analysis (TGA) was performed under O_2_ atmosphere from room temperature to 900 ^o^C at a heating rate of 10 ^o^C min^–1^.

**3. Electrochemical characterization**

The cathodes were prepared as follows: mixing active composites (I_2_@NC), super P, and polyvinylidene difluoride (PVDF) binder, at a weight ratio of 8:1:1 in *N*-methyl pyrrolidone (NMP) solvent by grinding and then coated on Ti foil (20 μm). After vacuum drying at 60 ℃, the loading of iodine in the electrode was about 1.0 mg cm^–2^. The coin cell CR2032 was assembled by the I_2_@NC as a cathode, Zn foil as an anode, Whatman glass fiber as a separator, and aqueous 2 M ZnSO_4_ as the electrolyte. Neware battery measurement system for constant current charge/discharge testing of cells in the voltage range of 0.6–1.6 V. Cyclic voltammograms (CV) and electrochemical impedance spectra (EIS) were conducted on a CHI 760E instrument.

**4. Adsorption Experiments**

To investigate the sustainability of the host material to iodine, the releasing test of iodine species was examined by adding I_2_, I_2_@S3-900, I_2_@S3-1000, I_2_@S3-1100 composites into the 2 M ZnSO_4_ electrolyte, respectively. In comparison with the obvious color change of electrolyte due to the dissociation of iodine, the color change was observed in the solutions after 1h, 4 h, 24 h, and 48 h, respectively.

In a 3 mL glass bottle, excessive iodine was added and stirred overnight. The saturated iodine was obtained by the supernatant rather than the precipitate. 10 mg of S3-900, S3-1000, and S3-1100 powders were immersed in saturated iodine solution, and the color changes were observed after 1h, 12 h, 24 h, and 48 h, respectively.

In a 3 mL glass bottle, iodine and zinc iodide (ZnI_2_) are mixed in a 2:1 molar ratio with 2 M ZnSO_4_ to obtain 4 mM Zn(I_3_)_2_ solution under constant stirring. 10 mg of S3-900, S3-1000, and S3-1100 powders were immersed in Zn(I_3_)_2_ solution, and the color changes were observed after 1h, 4 h, 24 h, and 48 h, respectively.

**5. Computational details**

All DFT calculations for periodic material systems were performed with the Vienna Ab initio simulation package (VASP).^[1]^ using the projector-augmented wave (PAW) method.^[2]^ The exchange–correlation function was handled using the generalized gradient approximation (GGA) formulated by the Perdew-Burke-Ernzerhof (PBE).^[3]^ The van der Waals (vdW) interactions are described with the DFT-D3 method in Grimme’s scheme.^[4,5]^ The interaction between the atomic core and electrons was described by the projector augmented wave method. The plane-wave basis set energy cutoff was set to 500 eV.^[6,7]^ The Brillouin zone was sampled with a 1 × 1 × 1 grid centered at the gamma (Γ) point for geometry relaxation. All the slabbed models possessed a vacuum spacing of ≈15 A˚ sampled, ensuring negligible lateral interaction of adsorbates.^[8]^ The bottom layers about half of the structure were kept frozen at the lattice position.^[9]^ All structures with a dynamic magnetic moment were fully relaxed to optimize without any restriction until their total energies were converged to < 1×10^-6^ eV,^[10]^ and the average residual forces were < 0.02 eV/Å.^[11,12]^

**6. Supplementary Figures**


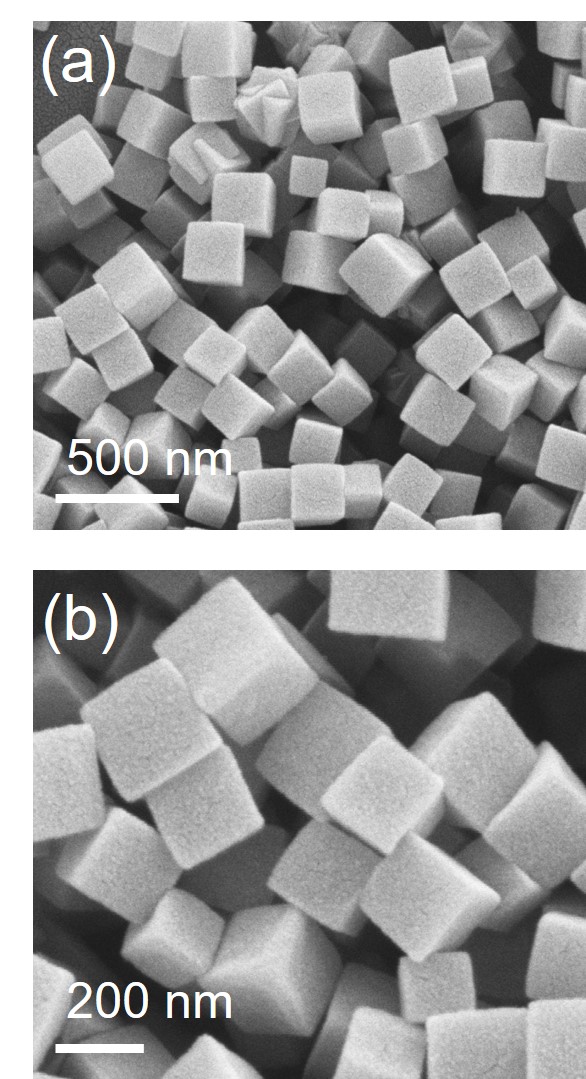


**Figure S1.** (a, b) SEM images of S3 .


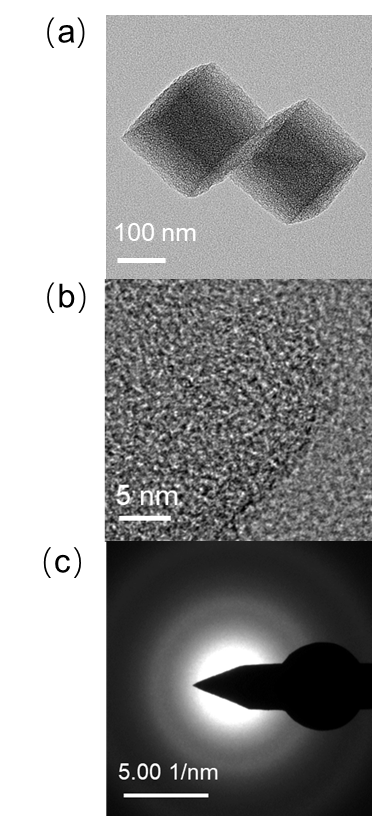


**Figure S2.** (a) TEM, (b) HRTEM and (c) SAED images of S3-1000.

**
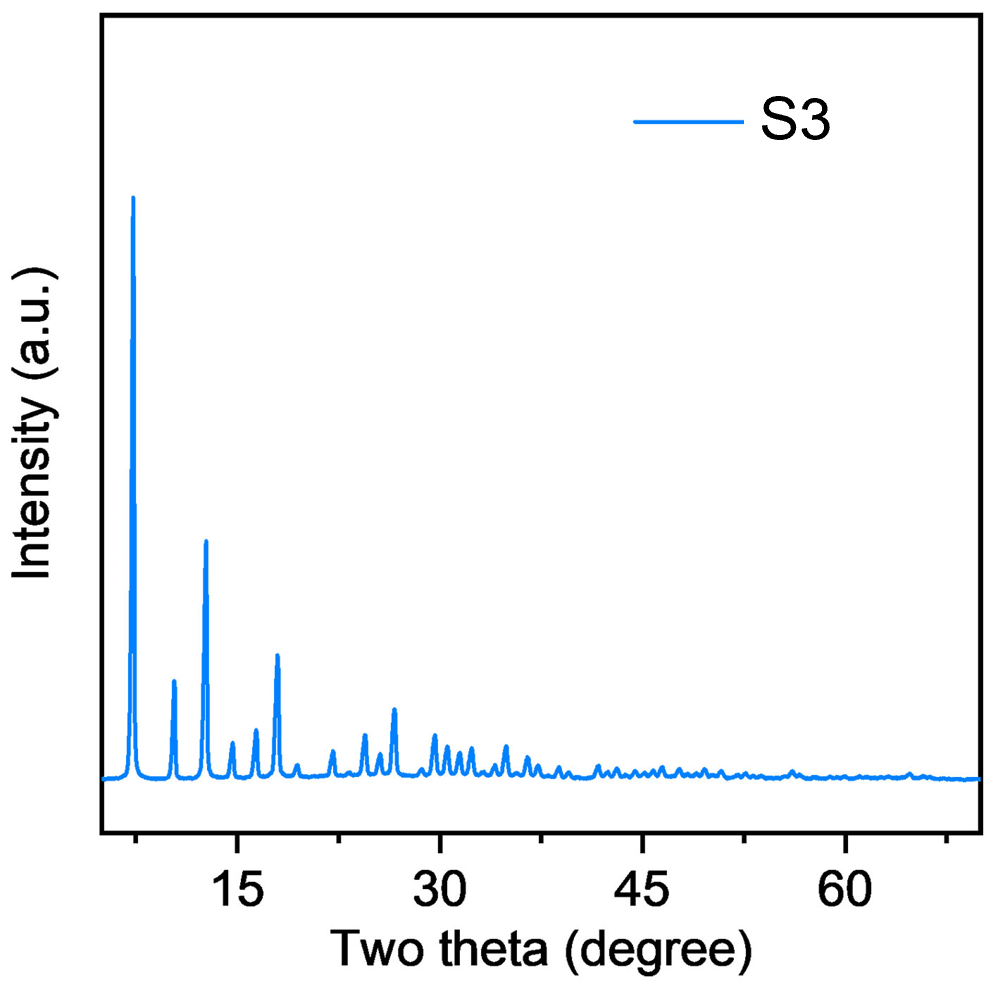
**

**Figure S3.** XRD pattern of the cubic S3 precursor.


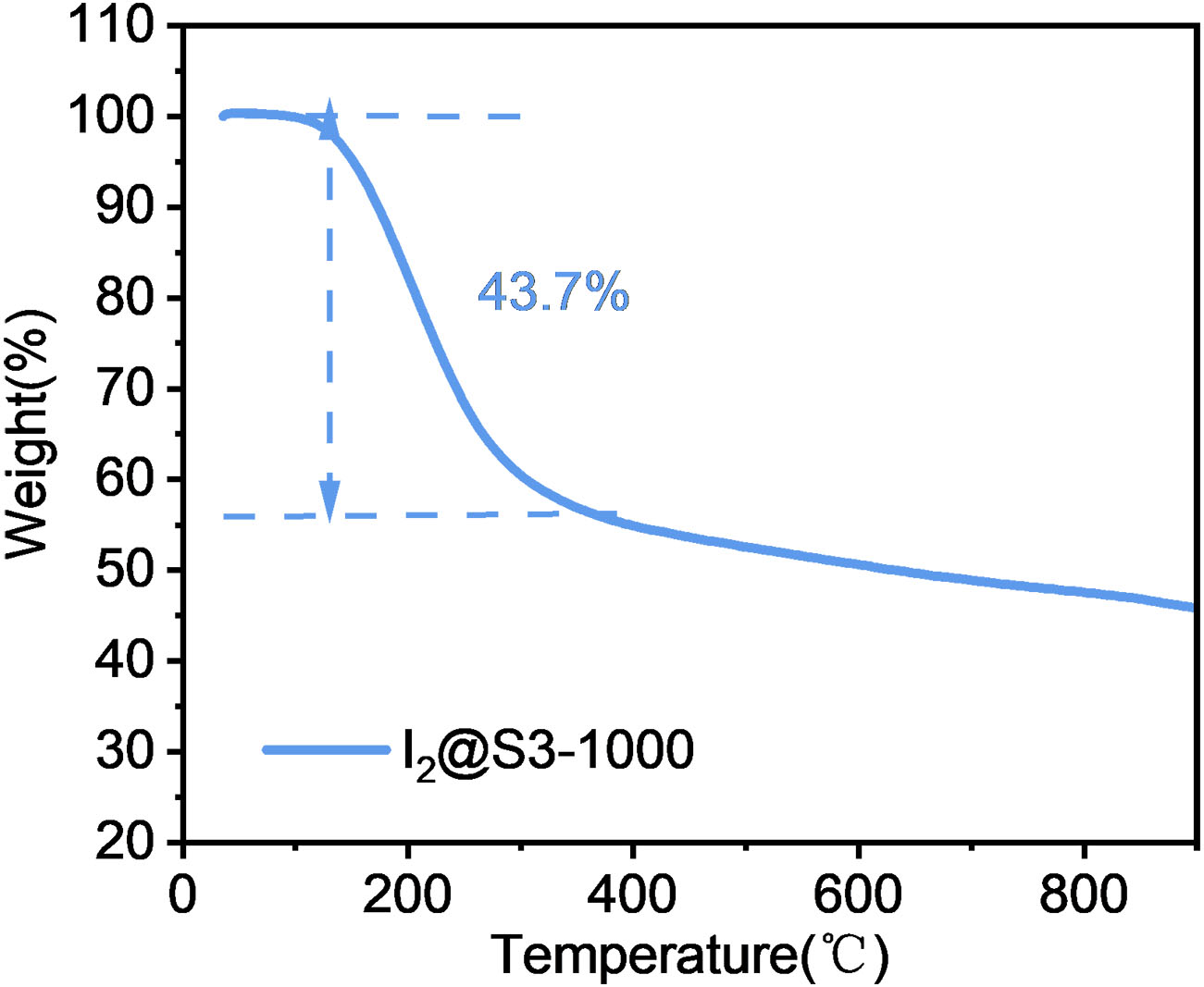


**Figure S4.** TGA curves of I_2_@S3-1000.


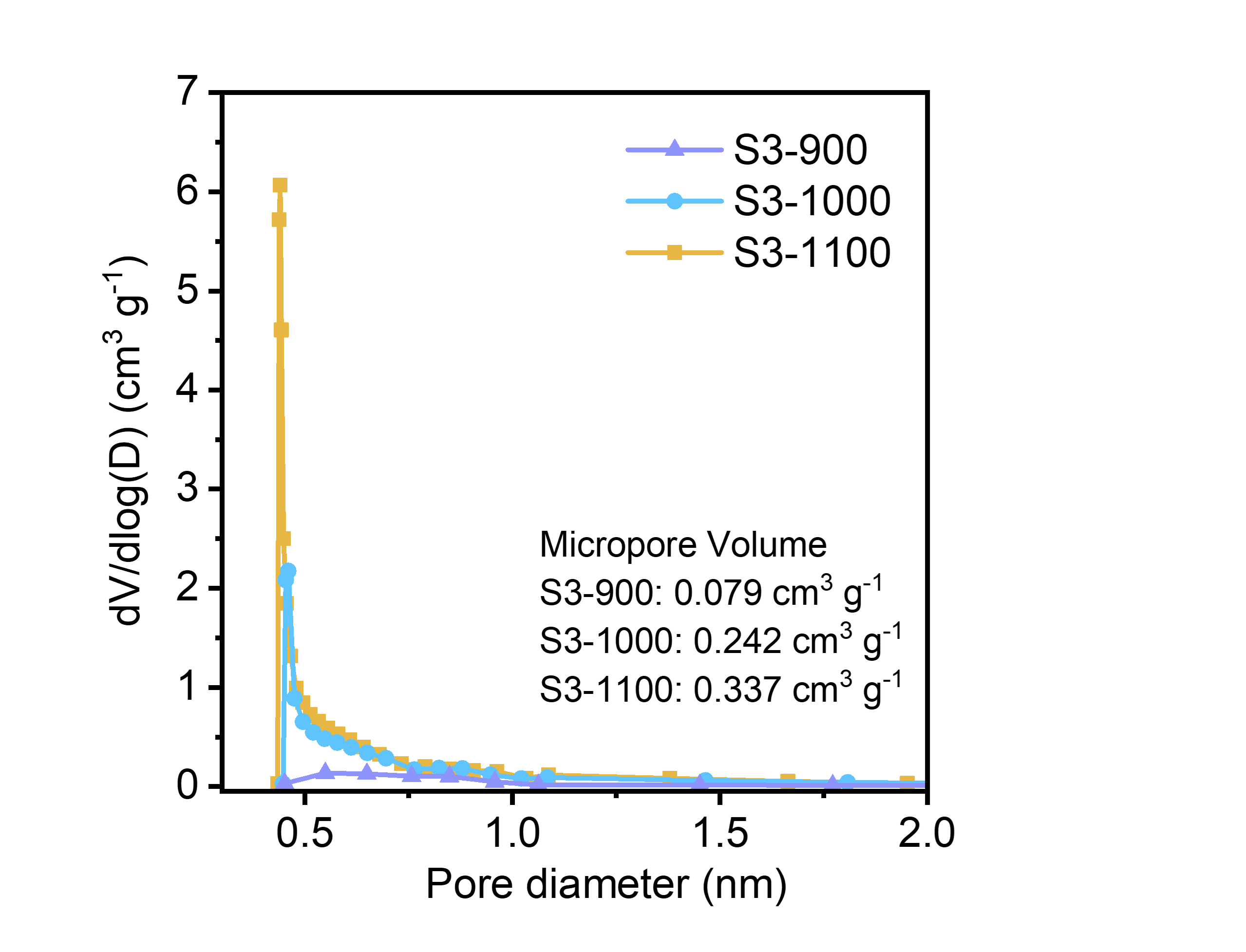


**Figure S5.** The micropore size distribution curves of S3-900, S3-1000 and S3-1100.


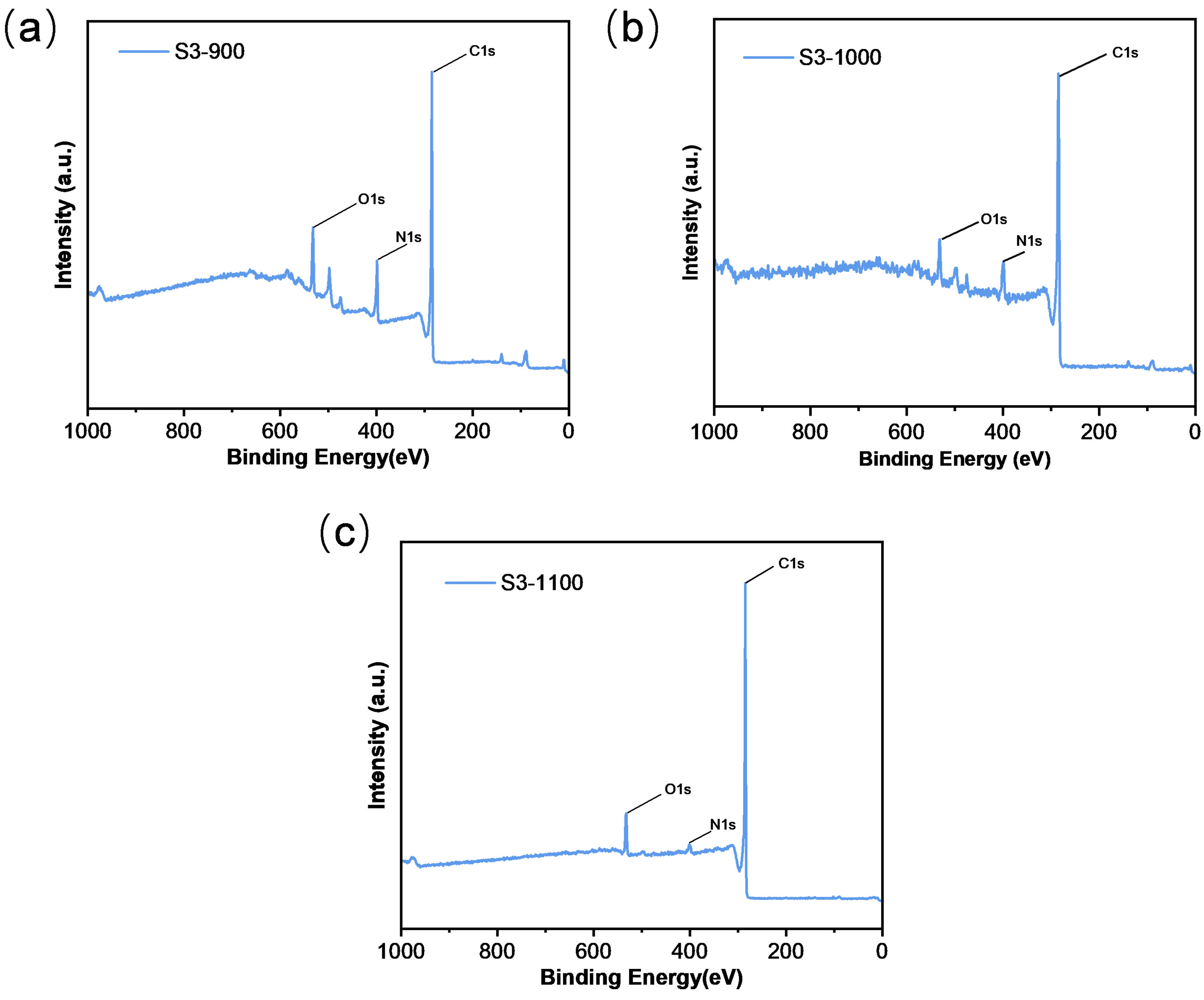


**Figure S6.** Full XPS spectrum of S3-X (X=900, 1000, 1100), respectively.

**
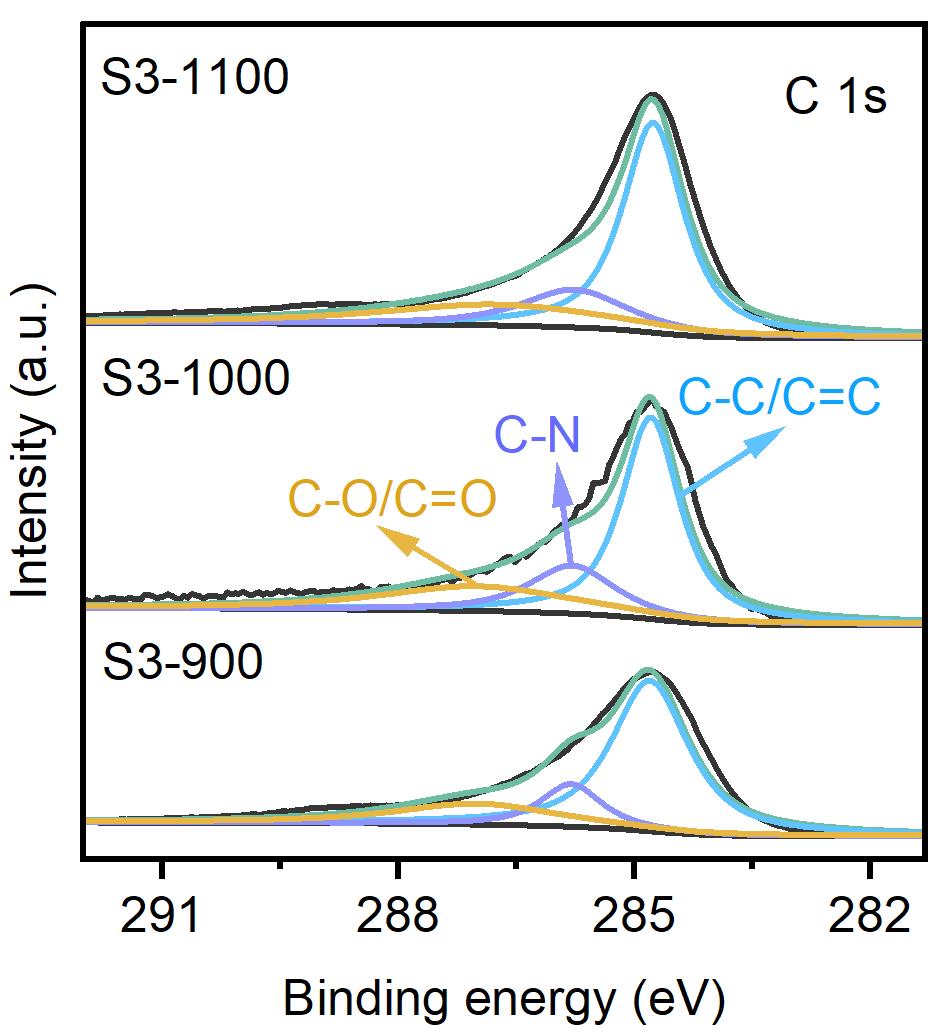
**

**Figure S7.** High resolution C 1s spectra of S3-900, S3-1000, and S3-1100, respectively.


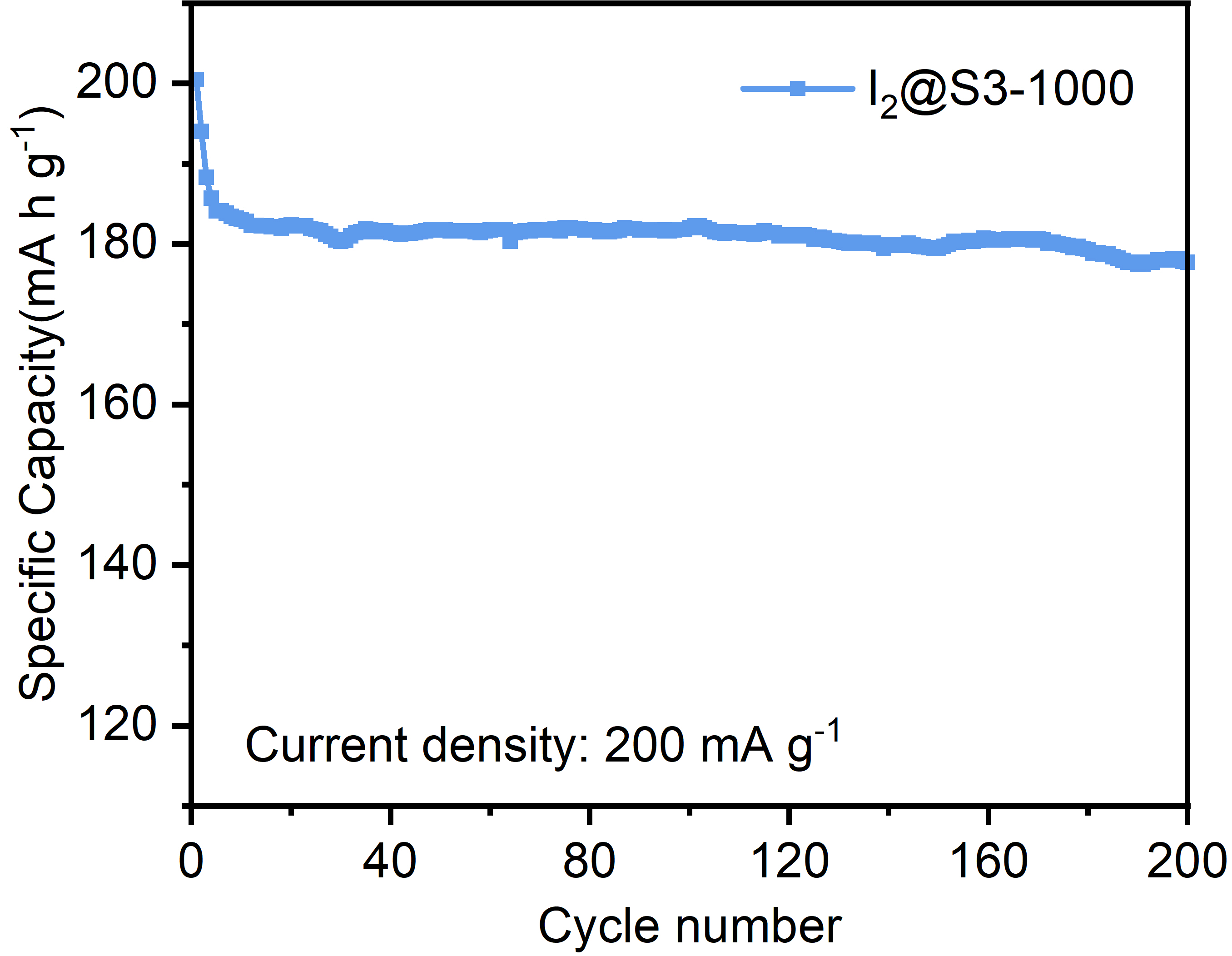


**Figure S8.** Cycling performance of I_2_@S3-1000 at 0.2 A g^-1^.


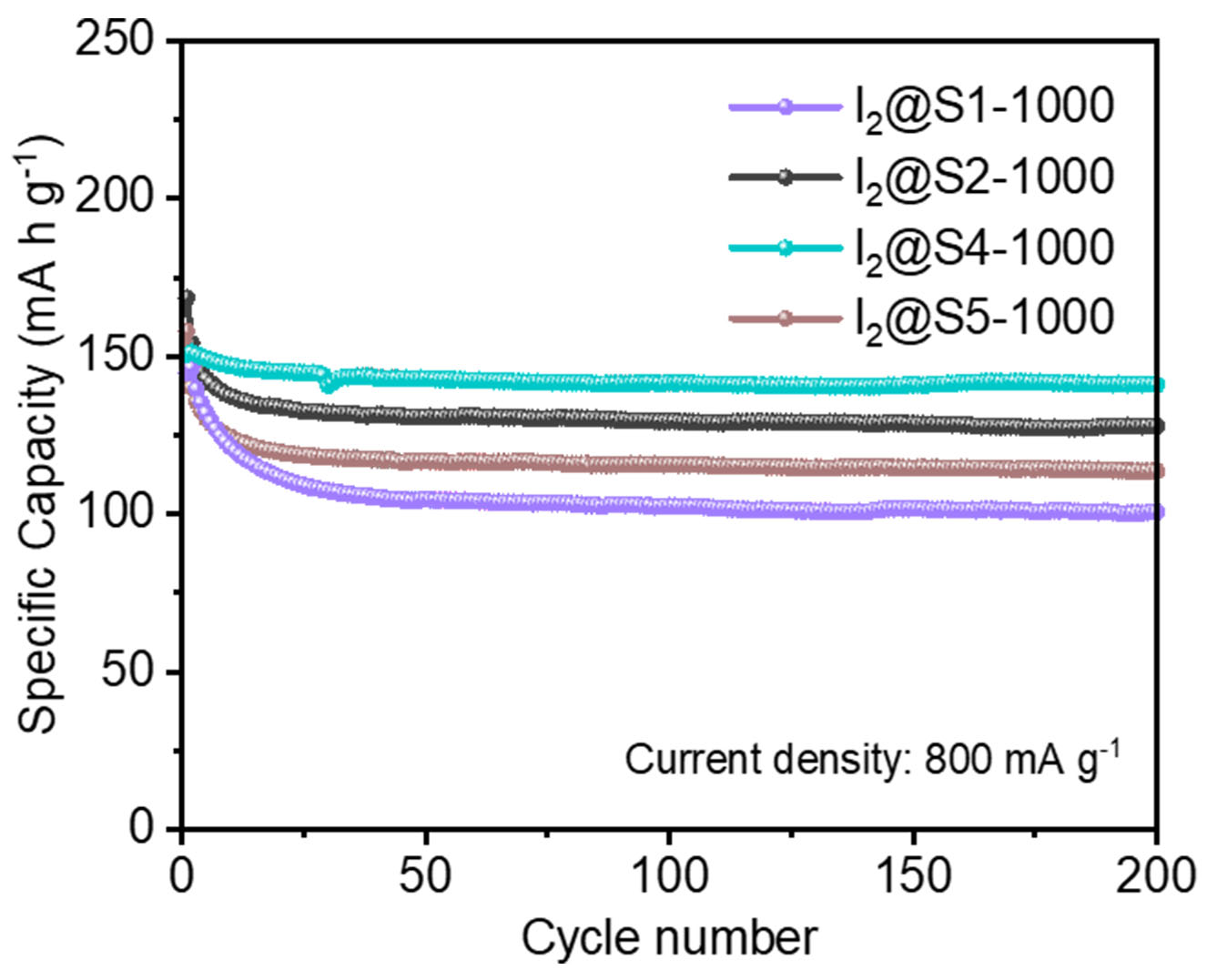


**Figure S9.** The cycling performance of the I_2_@S1-1000, I_2_@S2-1000, I_2_@S4-1000, and I_2_@S5-1000 cathodes at 800 mA g^–1^.


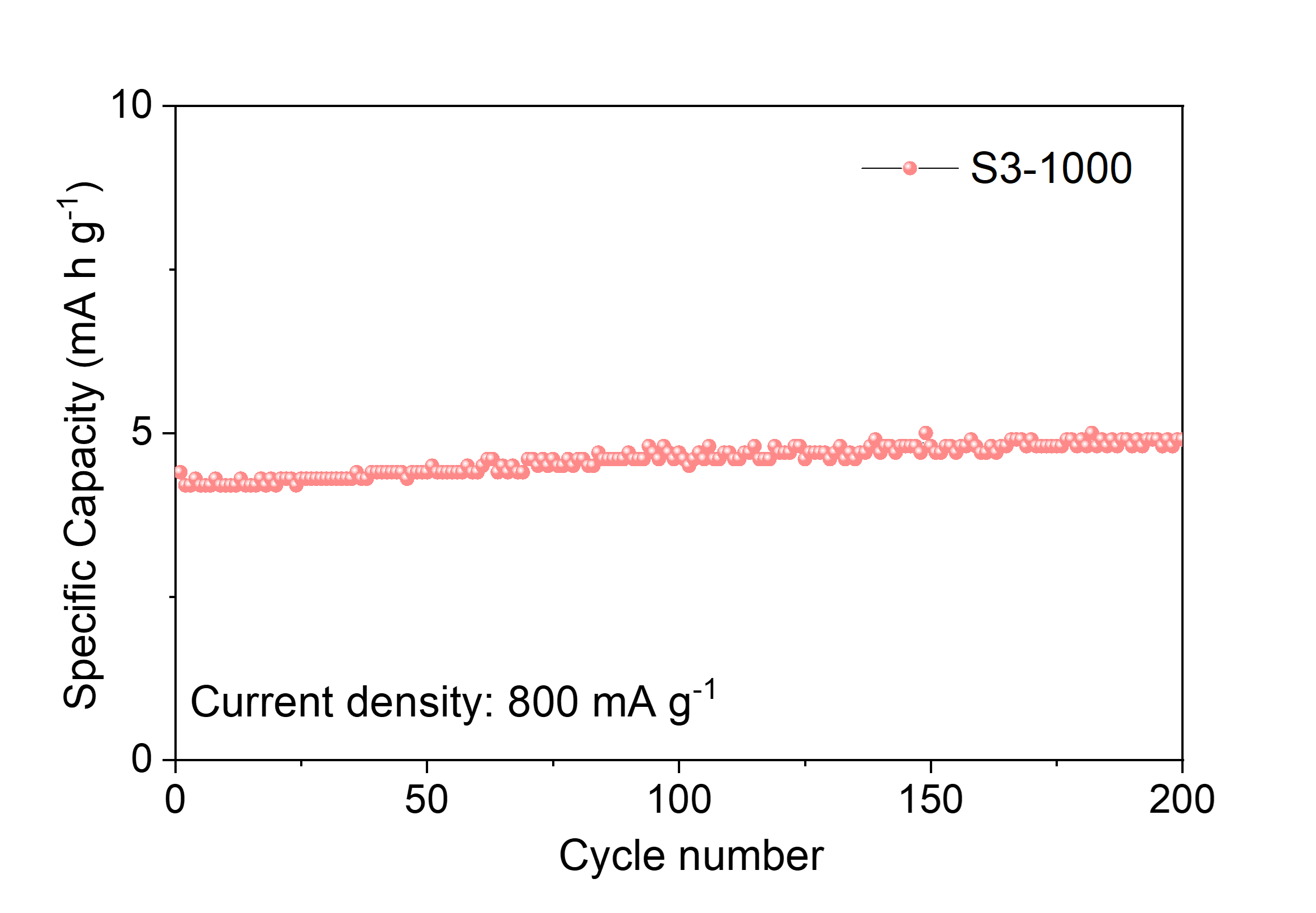


**Figure S10.** The cycling performance of the S3-1000 electrode at 800 mA g^–1^.


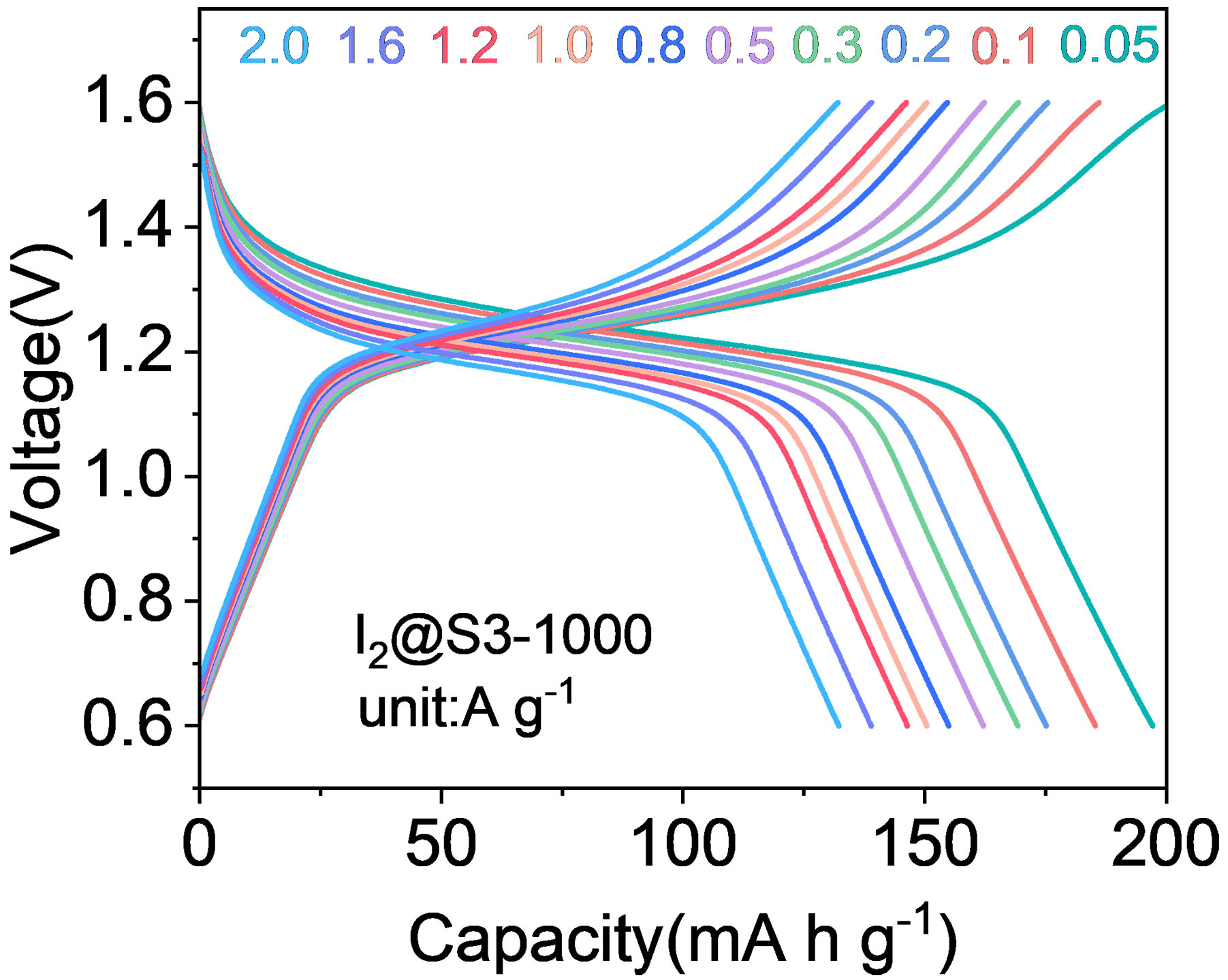


**Figure S11.** GCD curves of I_2_@S3-1000 at various current densities.


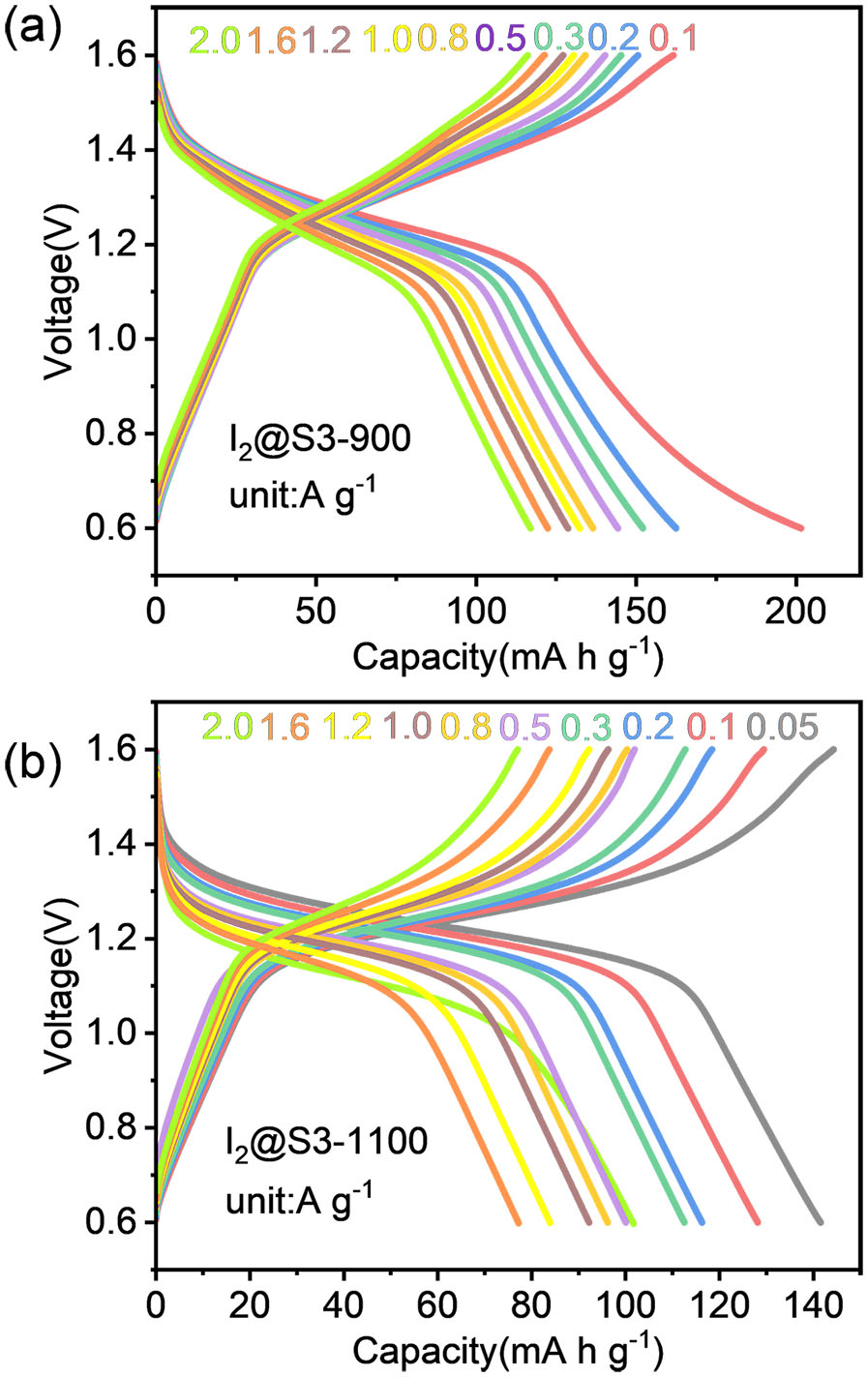


**Figure S12.** The GCD curves of I_2_@S3-900 and I_2_@S3-1100 at different current densities.


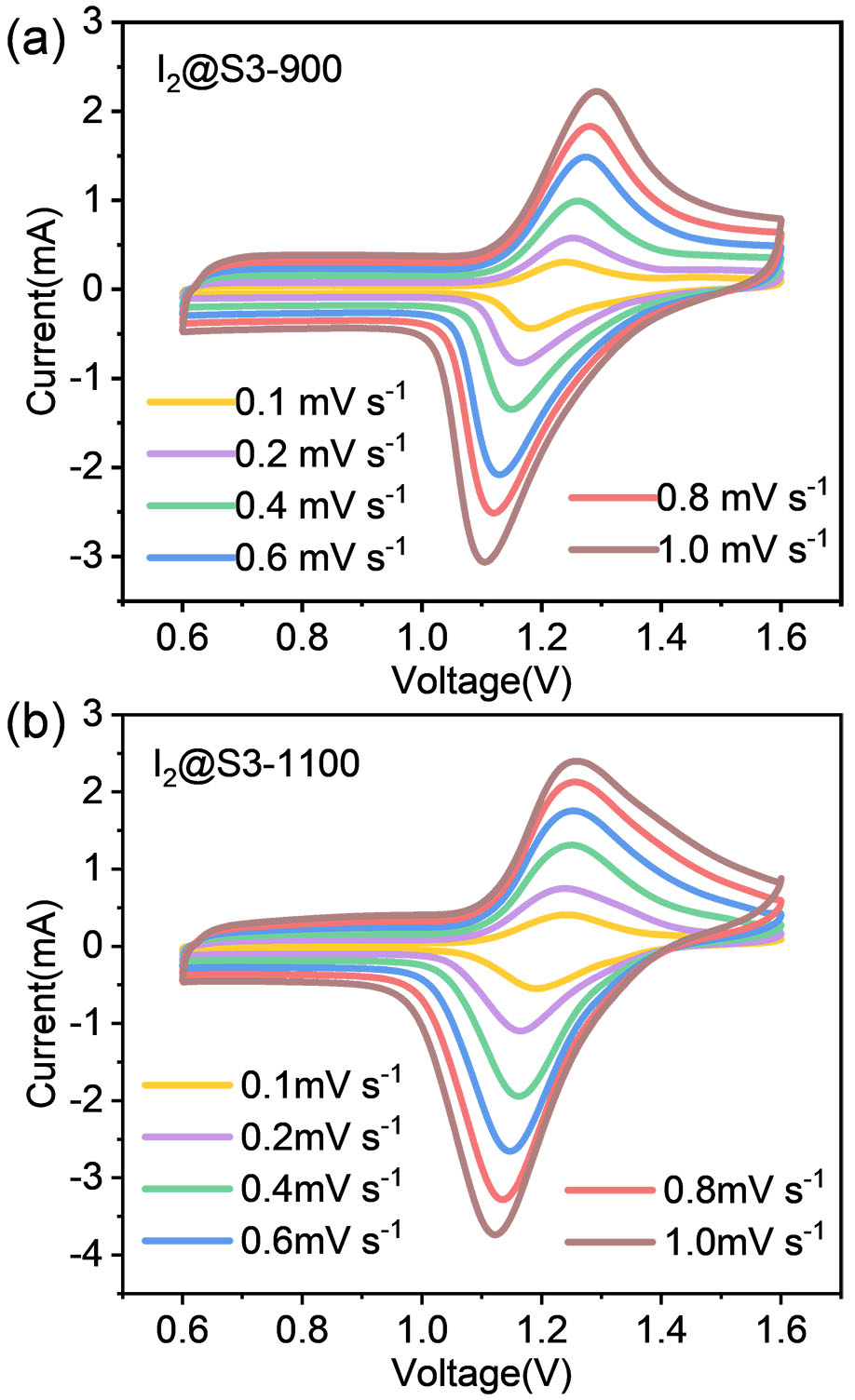


**Figure S13.** The CV curves of I_2_@S3-900 and I_2_@S3-1100 at different scan rates from 0.1 to 1.0 mV s^-1^.


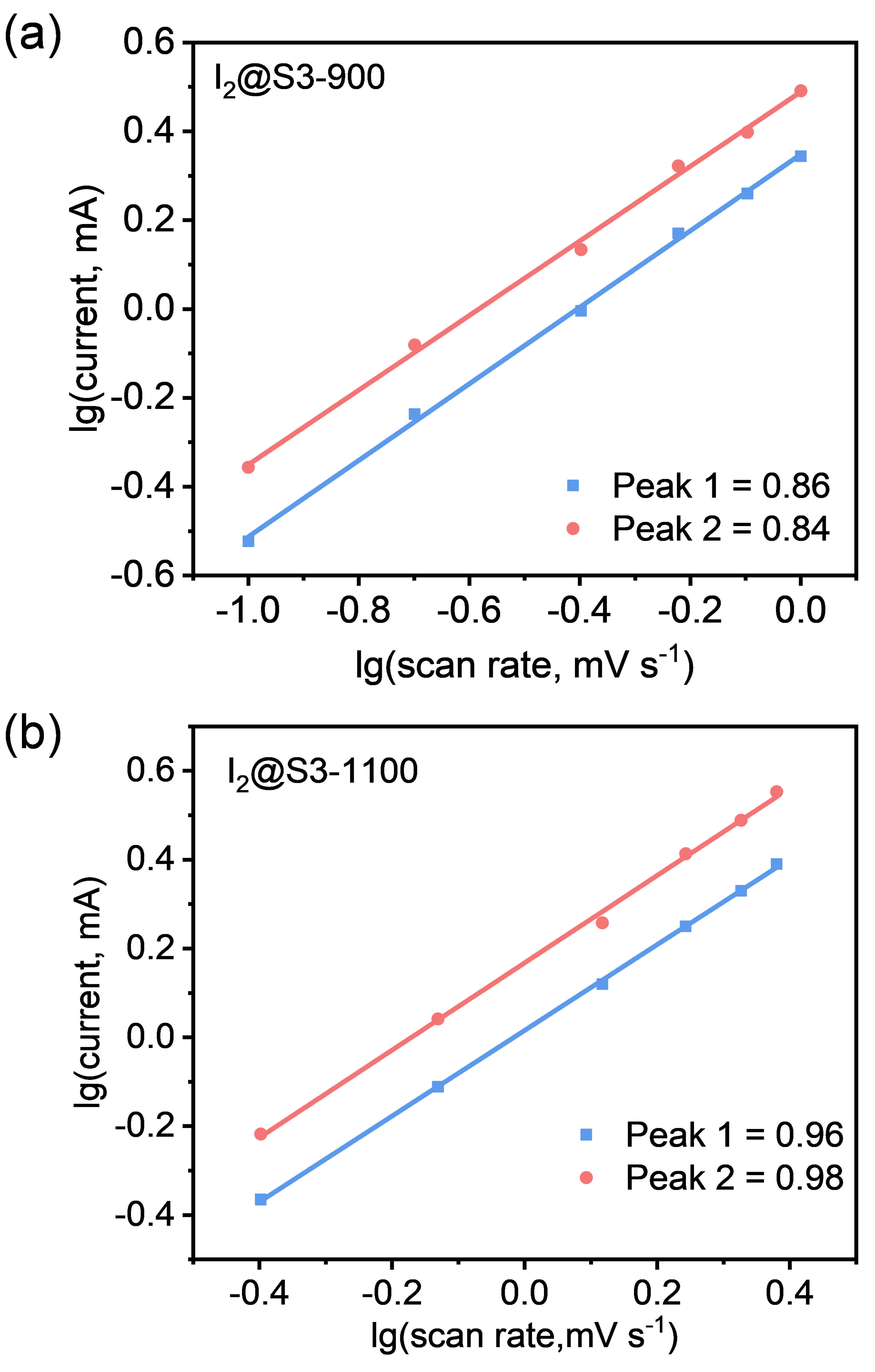


**Figure S14.** The linear fitting plots of the b values from CV curves of I_2_@S3-900 and I_2_@S3-1100, respectively.

**
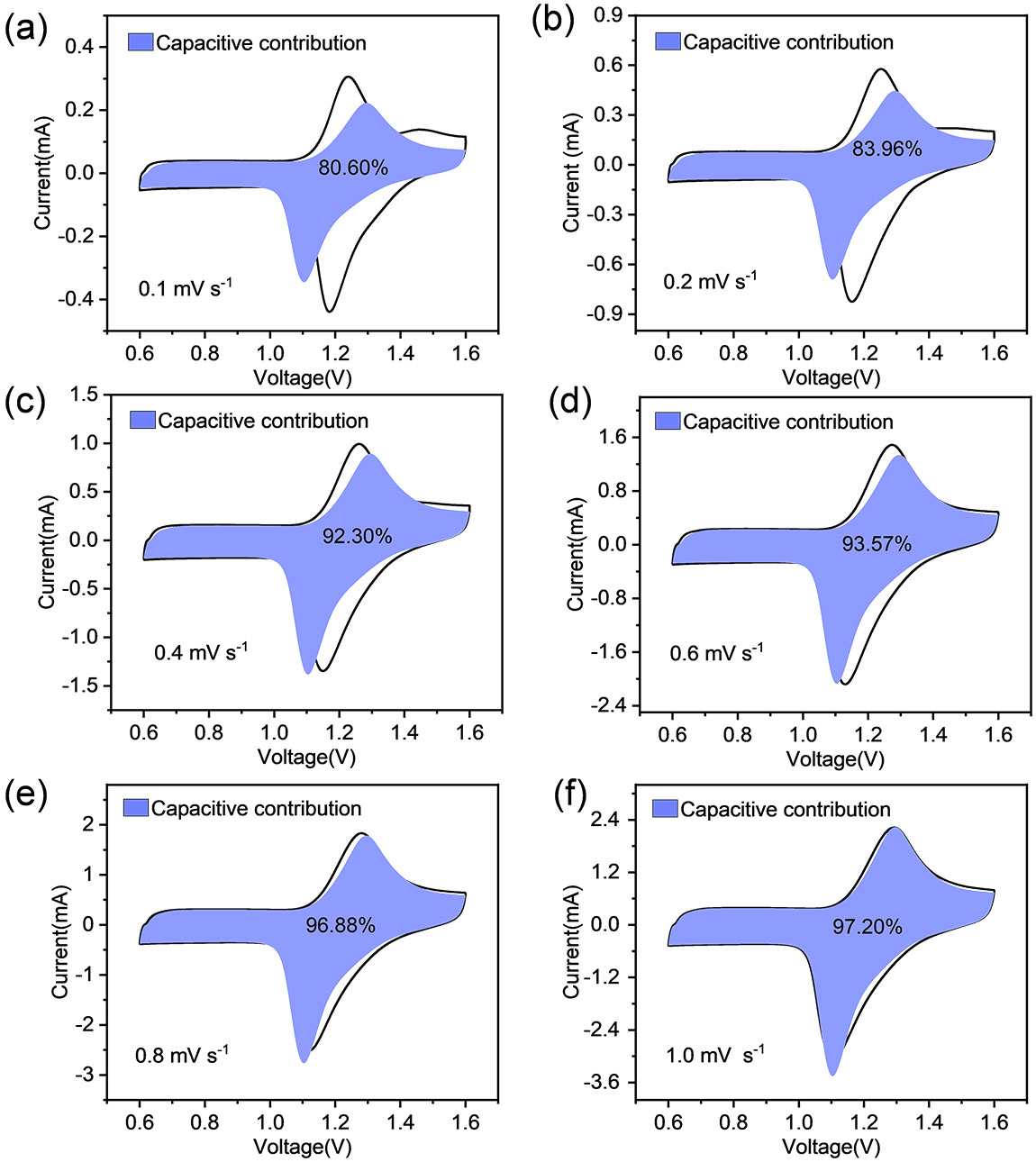
**

**Figure S15.** The capacitive contributions of I_2_@S3-900 at scan rates of 0.1, 0.2, 0.4, 0.6, 0.8 and 1.0 mV s^-1^.

**
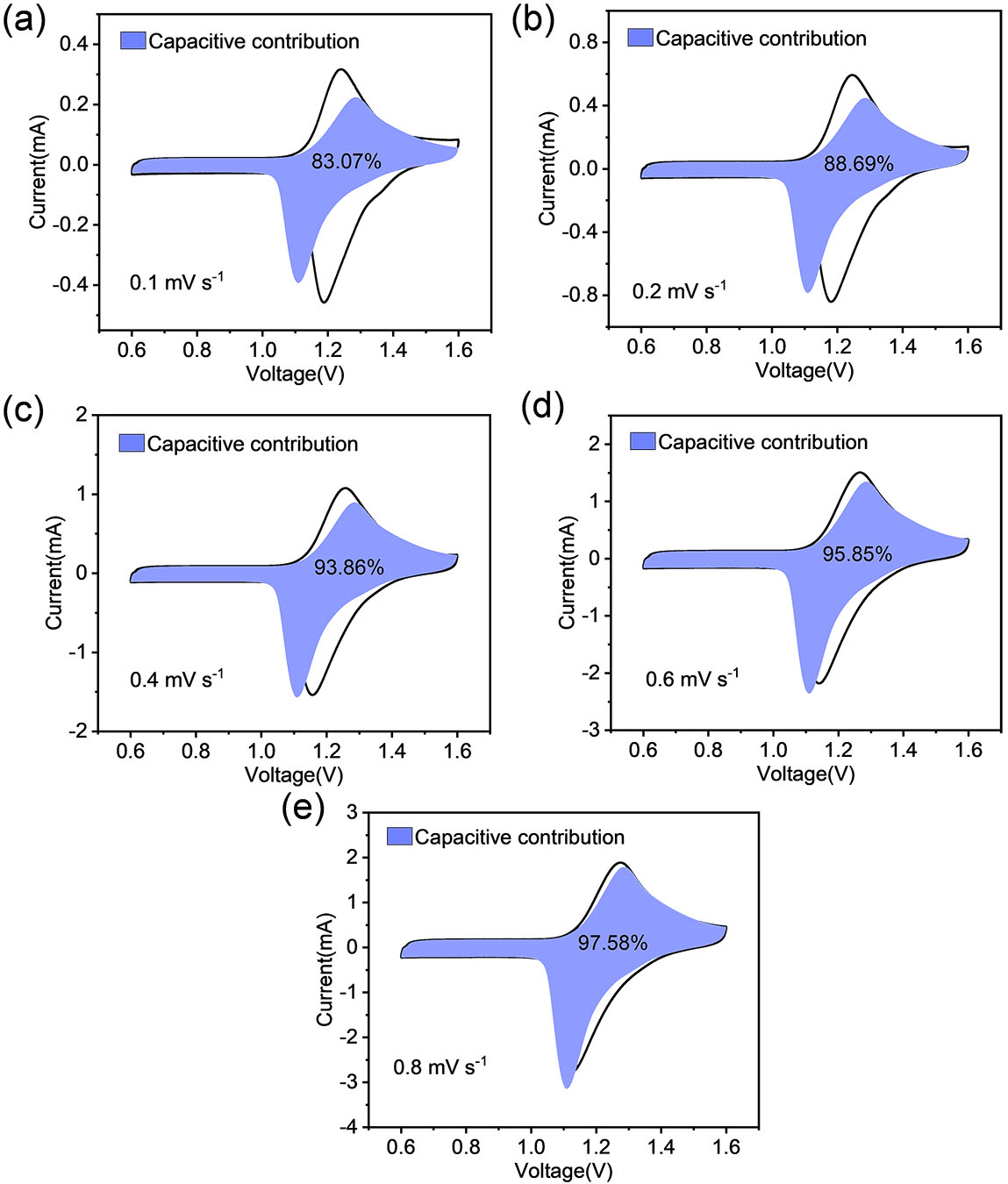
**

**Figure S16.** The capacitive contributions of I_2_@S3-1000 at scan rates of 0.1, 0.2, 0.4, 0.6 and 0.8 mV s^-1^.

**
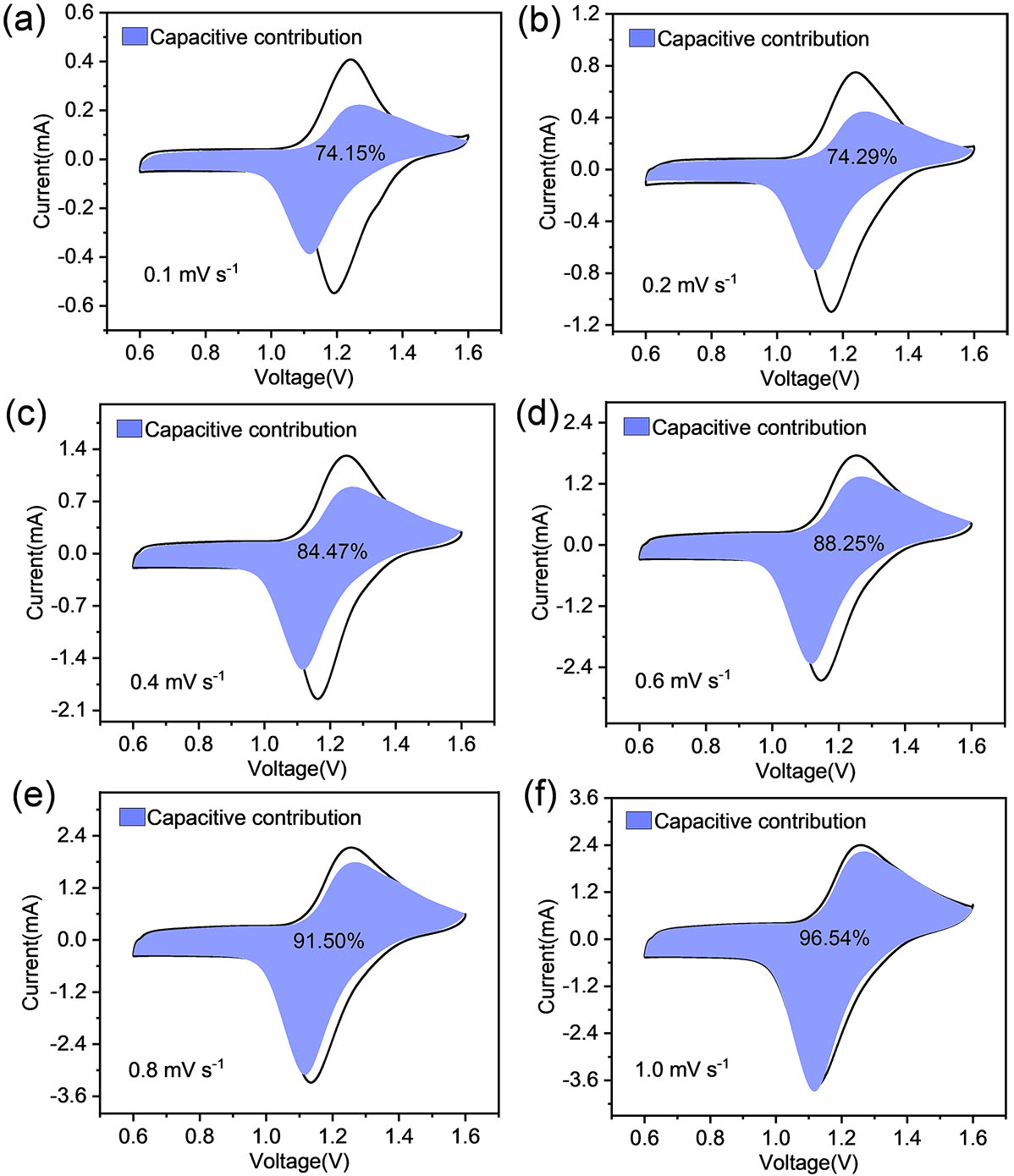
**

**Figure S17.** The capacitive contributions of I_2_@S3-1100 at scan rates of 0.1, 0.2, 0.4, 0.6, 0.8 and 1.0 mV s^-1^.

**7. Supplementary Tables**

**Table S1.** Comparison of different nitrogen species in XPS measurement.

| Samples | Pyridinic N | Pyrrolic N | Graphitic N |
| --- | --- | --- | --- |
| S3-900 | 52.01% | 13.53% | 34.46% |
| S3-1000 | 48.81% | 10.51% | 40.68% |
| S3-1100 | 17.03% | 5.84% | 77.13% |

**References**

[1] Kresse, G.; Furthmüller, J. *Comput. Mater. Sci.* **1996,** *6*, 15.

[2] Blöchl, P. E. Projector *Phys. Rev. B* **1994,** *50*, 17953.

[3] Ernzerhof, M.; Scuseria, G. E. *J. Chem. Phys.* **1999,** *110*, 5029.

[4] Grimme, S.; Antony, J.; Ehrlich, S.; Krieg, H. *J. Chem. Phys.* **2010,** *132*, 154104.

[5] Guan, D.; Xu, H.; Zhang, Q.; Huang, Y. C.; Shi, C.; Chang, Y. C.; Xu, X.; Tang, J.; Gu, Y.; Pao, C. W.et al. *Adv. Mater.* **2023,** *35*, 2305074.

[6] Xu, H. *Chem. Phys. Lett.* **2024,** *851*, 141492.

[7] Li, W.; Xu, H.; Zhang, H.; Wei, F.; Zhang, T.; Wu, Y.; Huang, L.; Fu, J.; Jing, C.; Cheng, J.et al. *Energy Environ. Sci.* **2023,** *16*, 4502.

[8] Zheng, Q.; Xu, H.; Yao, Y.; Dai, J.; Wang, J.; Hou, W.; Zhao, L.; Zou, X.; Zhan, G.; Wang, R.et al. *Angew. Chem. Int. Ed.* **2024,** *63*, e202401386.

[9] Guan, D.; Shi, C.; Xu, H.; Gu, Y.; Zhong, J.; Sha, Y.; Hu, Z.; Ni, M.; Shao, Z. *J. Energy Chem.* **2023,** *82*, 572.

[10] Xu, H.; Zhang, F.; Fang, L.; Xu, Y.; Yu, Z.-W.; Ma, L.; Guan, D.; Shao, Z. *Inorg. Chem.* **2024,** *63*, 19570.

[11] Xu, H.; Guan, D.; Ma, L. *Nanoscale* **2023,** *15*, 2756.

[12] Xu, H.; Zhu, J. Z.; Zou, C.; Zhang, F.; Ming, D.; Guan, D.; Ma, L. *Energy Fuels* **2023,** *37*, 16781.
